# Identifying heterogeneity using recursive partitioning: evidence from SMS nudges encouraging voluntary retirement savings in Mexico

**DOI:** 10.1093/pnasnexus/pgad058

**Published:** 2023-03-01

**Authors:** Avni M Shah, Matthew Osborne, Jaclyn Lefkowitz Kalter, Andrew Fertig, Alissa Fishbane, Dilip Soman

**Affiliations:** Department of Management, University of Toronto Scarborough, 1265 Military Trail, Toronto, ON, M1C 1A4, Canada; Rotman School of Management, University of Toronto, 105 St. George Street, Toronto, ON, M5S 3E6, Canada; Rotman School of Management, University of Toronto, 105 St. George Street, Toronto, ON, M5S 3E6, Canada; Department of Management, University of Toronto Mississauga, 3359 Mississauga Road, Mississauga, ON, L5L 1C6, Canada; Institute for Management and Innovation, University of Toronto, 3359 Mississauga Road, Mississauga, ON, L5L 1C6, Canada; ideas42, 80 Broad Street, 30th Floor, New York, NY 10004, USA; ideas42, 80 Broad Street, 30th Floor, New York, NY 10004, USA; ideas42, 80 Broad Street, 30th Floor, New York, NY 10004, USA; Rotman School of Management, University of Toronto, 105 St. George Street, Toronto, ON, M5S 3E6, Canada

**Keywords:** nudges, field experiment, retirement savings, machine-learning, heterogeneous treatment effects

## Abstract

Individuals regularly struggle to save for retirement. Using a large-scale field experiment (N=97,149) in Mexico, we test the effectiveness of several behavioral interventions relative to existing policy and each other geared toward improving voluntary retirement savings contributions. We find that an intervention framing savings as a way to secure one’s family future significantly improves contribution rates. We leverage recursive partitioning techniques and identify that the overall positive treatment effect masks subpopulations where the treatment is even more effective and other groups where the treatment has a significant negative effect, decreasing contribution rates. Accounting for this variation is significant for theoretical and policy development as well as firm profitability. Our work also provides a methodological framework for how to better design, scale, and deploy behavioral interventions to maximize their effectiveness.

**Significance Statement**

Practitioners and researchers have increasingly sought out behavioral interventions to help people save for retirement. To be effective, these interventions should scale to broad populations and across different cultural groups and produce long-lasting effects. Using a field experiment, we test the impact of various behavioral interventions on increasing voluntary retirement savings contributions. We find that framing savings as a way to secure one’s family future significantly increases contributions. Leveraging recursive partitioning techniques, we identify subgroups with lower- or higher-than-average treatment effects. Identifying this subgroup heterogeneity has significant substantive implications when scaling the intervention broadly. Our work provides a methodological framework for better designing, scaling, and deploying behavioral interventions to maximize their effectiveness.

Individuals regularly struggle to save for retirement. Many obstacles, such as a lack of planning, not knowing how much to save, exhibiting a present bias, and underestimating future needs, prevent individuals from making retirement contributions and securing their financial future. To overcome these hurdles, practitioners and researchers have increasingly sought out behavioral interventions (1–5). To be effective, these interventions should scale to broader populations and produce long-lasting effects. This raises a few key questions: What interventions will help people save for retirement? Will an intervention motivate all people similarly, particularly when implemented “at scale” (6, 7)? Will the effectiveness of these interventions be short-lived or will they persist over time?

In this work, we partnered with a Mexican bank and use a highly powered field experiment in Mexico (N=97,149)—a country where less than 0.5% make a voluntary savings contribution annually (8)—to investigate the effectiveness of several behavioral interventions designed to improve voluntary retirement contributions. We leverage previously successful interventions (i.e. basic reminder alerts, pennies-a-day/small amount framing, fresh start appeals, concrete visualization of one’s individual future) and explore whether redesigned and simplified account statements and SMS reminders improve voluntary savings relative to sending the status quo, standard account statement. In addition to these previously effective interventions, we test an under researched and previously undocumented intervention in the domain of financial savings—framing the savings decision as a way to secure their family’s financial future. We compare the effectiveness of this Family Security SMS relative to the status quo account statement and relative to the other behavioral interventions.

We observe a main, average treatment effect of the Family Security SMS intervention relative to both the status quo account statement as well as relative to the other intervention conditions. The Family Security SMS significantly increases voluntary savings contributions by roughly 45% relative to the standard account statement and increases contributions by between 18–51% relative to the other behavioral interventions. However, prior work has shown that average treatment effects can potentially mask underlying heterogeneity (9, 10). As interventions scale and are replicated across different behavioral and cultural contexts, it becomes difficult for researchers to have clear expectations for where heterogeneity may exist. While missing unexpected effects between variables may not be as consequential in smaller population samples, the ability to accurately detect treatment heterogeneity—and make out-of-sample predictions to avoid the perils of overfitting—is increasingly useful for policymakers and practitioners when the goal is to scale interventions more broadly (9, 11–13). Evidence suggests that data-driven methods, such as recursive partitioning, may reveal subpopulations with lower-than-average or higher-than-average treatment effects (9, 10).

To assess whether specific subgroups respond heterogeneously to the various interventions, we explore whether the Family Security SMS is effective across subpopulations using two recursive partitioning approaches, Causal Tree and Causal Forest. We find evidence of significant heterogeneity: The effectiveness of the Family Security SMS is highly dependent on age and gender. Compared to the status quo policy, sending a Family Security SMS reminder improves contribution rates by 88% for people between the ages of 29–41 (two-tailed unadjusted P<0.001) but leads to a 54% backfire for those below the age of 29 (two-tailed unadjusted P<0.001). The backfire of the Family Security SMS, as well as all behavioral interventions, is particularly pronounced for women. Notably, we find no age and gender-based heterogeneity using an OLS regression with interactions between treatment, gender, and various age specifications. These results highlight the benefits of using data-driven approaches to identifying heterogeneous treatment effects especially in cases when researchers may not have prior expectations of effects.

Identifying this heterogeneity has significant policy implications for scaling the intervention to larger populations. A blanket, one-size fits all approach would result in a 44% increase in new contributors per quarter relative to the firm’s standard account statement. In contrast, a segmented application of this intervention targeting the intervention only to those above the age of 29 would result in a 61% increase in contributors relative to the control and 38% increase relative to a blanket, one-size-fits all approach.

This paper makes several contributions. First, we demonstrate the efficacy of a family oriented message to motivate individuals to save for retirement. Second, we show the benefits of using recursive partitioning techniques in large-scale field experiments to better identify treatment heterogeneity. While we observe a positive, average treatment effect of the Family Security SMS, we show that it is not a viable solution for all subgroups. The magnitude and direction of the Family Security SMS intervention, as well as other behavioral interventions, can vary substantially across subpopulations. Our work builds on prior work (9, 14) by providing a way to better design, scale, and deploy behavioral interventions to maximize their effectiveness. Finally, we show that easy to implement, low-cost SMS reminders that focus on the benefits of saving for one’s family can have persistent effects on retirement savings. We show that the Family Security SMS improves contribution rates for certain subgroups several months after the intervention. Our work responds directly to the calls for better tailoring, targeting, and customizing behavioral interventions based on the circumstances of a subpopulation, rather than using a one-size-fits-all approach (7, 15, 16).

In what follows, we present a brief background on our empirical setting as well as our experimental procedure. We then present the main results, and identify heterogeneity using recursive partitioning via Causal Tree and Causal Forest. We look at the implications resulting from each method and discuss the impact on scaling interventions based on each method. We end by investigating whether our interventions show evidence of persistence by looking at the effects in the period following our experiment, and conclude.

## Results

Our field experiment used a representative sample of 97,149 active customers drawn from the firm’s roughly two million customers. Overall, our sample had an average age of 33.8 years, 58% were male, and 0.61% made a prior voluntary contribution. As shown in Table 1, study arms were well-balanced on age, gender, and retirement contribution history (*P* values from all *F* tests >0.05, two-tailed).

**Table 1.** Summary statistics and balance table of pretreatment and demographic variables.

*Condition*	*N*	Made a Prior contribution	Prior contribution amount ($) *(IHS transformed)*	Male	Mean age
Standard account statement (Control)	13,902	0.00604	0.05363	0.57524	33.79
		(0.07750)	(0.70155)	(0.49432)	(5.85)
New statement (No SMS)	13,875	0.00605	0.05688	0.57759	33.75
		(0.07757)	(0.73834)	(0.49396)	(5.82)
New statement + Basic Alert SMS	13,859	0.00664	0.06158	0.57565	33.74
		(0.08121)	(0.76570)	(0.49426)	(5.82)
New statement + Fresh Start SMS	13,882	0.00656	0.05956	0.58587	33.78
		(0.08070)	(0.74566)	(0.49259)	(5.79)
New statement + Small Amounts SMS	13,877	0.00584	0.05087	0.57700	33.72
		(0.07618)	(0.67626)	(0.49405)	(5.83)
New statement + Individual Goals SMS	13,901	0.00468	0.04295	0.58478	33.73
		(0.06822)	(0.63494)	(0.49278)	(5.79)
New statement + Family Security SMS	13,853	0.00671	0.06382	0.58103	33.71
		(0.08166)	(0.78641)	(0.49341)	(5.82)
*P*-value of regression *F*		0.24703	0.17744	0.37188	0.89144

This table describes the sample in each experimental condition across the two pretreatment control variables and two demographic variables, comparing treatment and control groups across variables. Made a Prior Contribution is a dummy variable indicating if a voluntary retirement contribution was made during the pretreatment period (February 22 to September 26). Total prior contribution amount applies an inverse hyperbolic sine transformation to the total amount contributed to the voluntary retirement savings account during the pretreatment period (February 22–September 26). Male is a dummy variable where 1 indicates individuals identifying as Male and 0 indicates identifying as Female. Standard deviations are reported in parentheses. Statistical significance is indicated by *, **, and *** for 10%, 5%, and 1% *P*-value, respectively, using a two-tailed test.

We measured the proportion of people who made a voluntary savings contribution and the amount contributed from the time that the retirement account statements and first SMS messages were sent (October 3) to the day before the next statement was sent (December 31). We provide a visualization of the experimental timeline and outcomes measured in Fig. S1 of the Supplemental Materials.

**Fig. 1.**
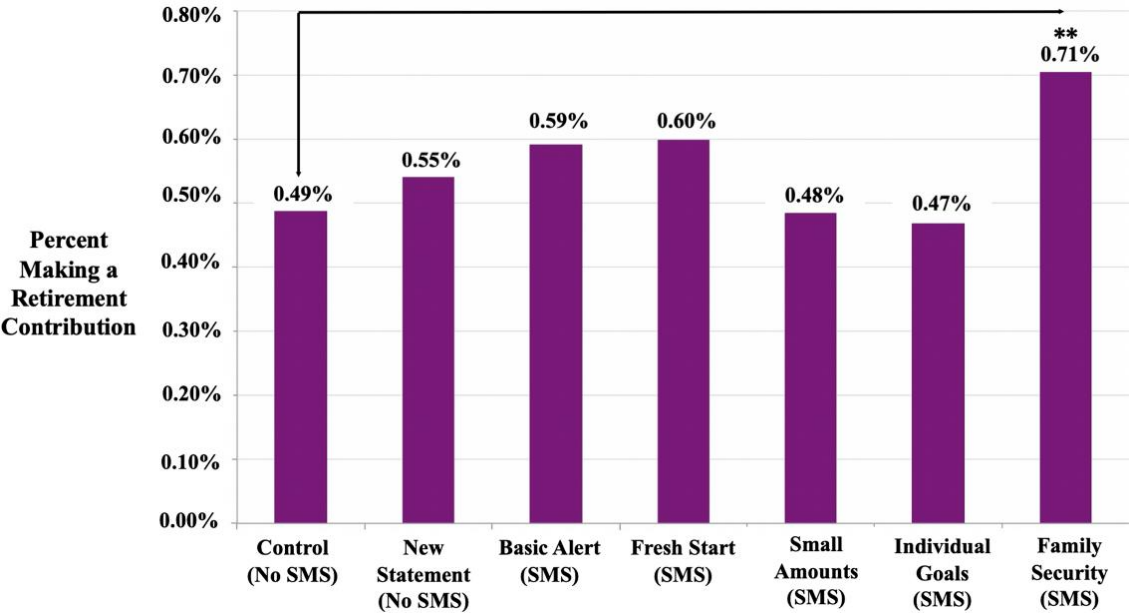
Proportion making a retirement contribution, by condition. This figure illustrates the proportion making a retirement contribution as a function of the experimental condition. Significance is indicated by *, **, and *** at 10%, 5%, and 1% for a two-tailed test, respectively and is based on a comparison between the treatment and the control group where individuals received the standard account statement.

**Fig. 2.**
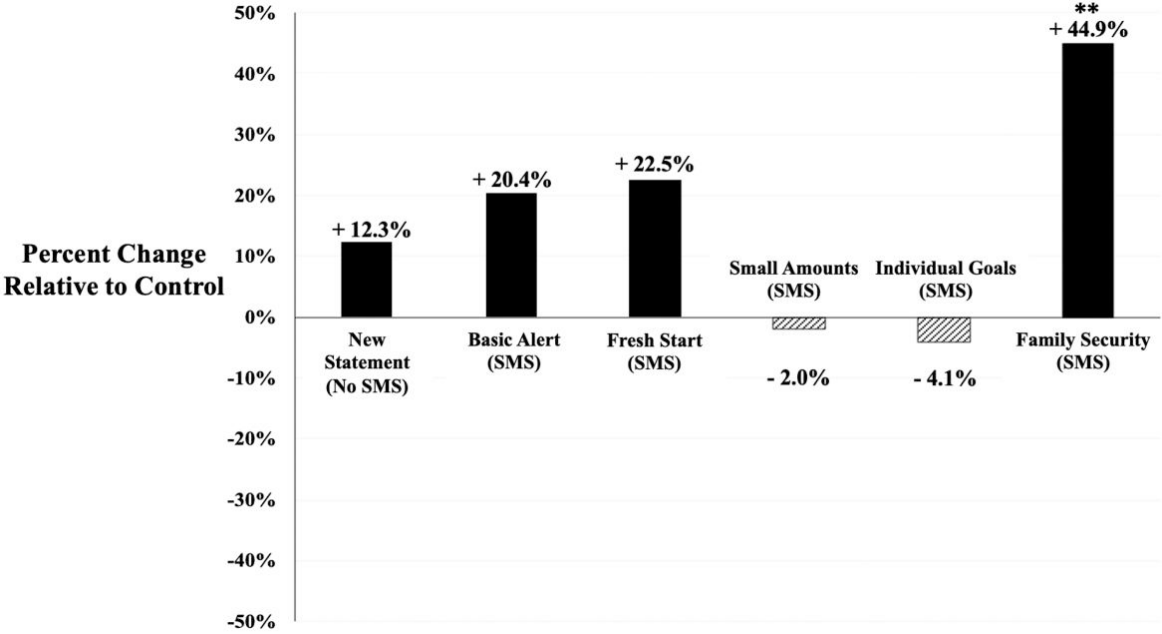
Percent change in contribution likelihood relative to control. This figure depicts the percent change in contribution likelihood comparing each intervention relative to the standard account statement. Significance is indicated by *, **, and *** at 10%, 5%, and 1% for a two-tailed test, respectively and is based on a comparison between the treatment and the control group where individuals received the standard account statement.

When just the standard account was sent—the baseline condition—we observe that 0.49% of individuals made a voluntary retirement contribution. As Fig. 1 shows, four of our six interventions directionally increased contribution rates. However, only the Family Security SMS, which framed retirement savings as a way to “secure your future and that of your family today,” followed by a reminder text that “it’s never too late to secure a better future for you and your family. Contribute to your Afore account,” significantly improved voluntary retirement contributions relative to the standard account statement (two-sided unadjusted P=0.018). In Fig. 2, we show that for the cost of sending two text messages (less than $0.25 USD), the Family Security SMS boosted contributions by about 45% relative to the standard account statement.

To estimate average treatment effects, we further control for multiple comparisons and use pretreatment controls (i.e. prior contribution rates). Recent research has suggested that the inclusion of pretreatment outcomes may improve statistical power (17, 18). We use the following ANCOVA specification estimating our effects, both with and without the inclusion of prior contribution history:


(1) $$Y_{i1}=\gamma_{0}+\sum_{\;j=1}^{6}\gamma_{j}\;Treat_{ij}+\theta {\bar{Y}}_{i0}+\varepsilon_{it},$$

where $Treat_{ij}$ is a dummy variable that is 1 if individual *i* receives treatment *j*, while ${\bar{Y}}_{i0}$ indicates a binary variable for whether or not individual *i* contributing in the eight months prior to experimental period *j*. The constant $\gamma_{0}$ measures the conditional mean of $Y_{i1}$ in the control group (in other words, treatment j=0 is the control). For contribution likelihood, $Y_{i1}$ is a binary variable equal to 1 if individual *i* made a contribution during our time period of interest. For contribution amount, we apply an inverse hyperbolic sine transformation to $Y_{i1}$ to account for skew, while still retaining values of zero if individual *i* did not make a contribution during our experimental period (19, 20).

Our main analyses compare each intervention on contribution likelihood and contribution amount using the status quo, standard account statement as the baseline condition given that this is what all account holders receive prior to our interventions and following the conclusion of the experiment. In Table 2, we find that only the Family Security SMS produced a positive and statistically significant boost in our two outcome variables of interest, inclusive of pretreatment controls for prior contribution (Contribution likelihood: β=0.00164, SE=0.00047, P<0.001, two-tailed; Contribution Amount: β=0.01526, SE=0.00405, P<0.001, two-tailed). To test for robustness, we find that the Family Security SMS is the only intervention to produce a significant positive effect when using a log-transformation for contribution amount at the P<0.05 level, two-tailed (see Table S3 in the Supplemental Materials).

**Table 2.** Voluntary savings contribution likelihood and amount, by treatment.

Dependent variable:	Contribution likelihood			Contribution amount ($) *(IHS transformed)*		
*Condition*	Mean	(1)	(2)	Mean	(3)	(4)
Standard statement *(Baseline)*	0.49%	–	–	$114.98	–	–
New statement (No SMS)	0.55%	0.00066	0.00065	$124.47	0.00674	0.00666${}^{*}$
		(0.00086)	(0.00044)		(0.00732)	(0.00371)
New statement + Basic Alert SMS	0.59%	0.00103	0.00054	$165.28	0.00933	0.00527
		(0.00088)	(0.00048)		(0.00741)	(0.00406)
New statement + Fresh Start SMS	0.60%	0.00109	0.00067	$105.62	0.00924	0.00574
		(0.00088)	(0.00045)		(0.00738)	(0.00378)
New statement + Small Amounts SMS	0.48%	− 0.00006	0.00010	$91.08	− 0.00079	0.00061
		(0.00083)	(0.00045)		(0.00695)	(0.00384)
New statement + Individual Goals SMS	0.47%	− 0.00022	0.00089${}^{**}$	$80.20	− 0.00287	0.00646${}^{*}$
		(0.00083)	(0.00045)		(0.00682)	(0.00369)
New statement + Family Security SMS	0.71%	0.00218${}^{**}$	0.00164${}^{***}$	$182.11	0.01984${}^{**}$	0.01526${}^{***}$
		(0.00093)	(0.00047)		(0.00786)	(0.00405)
Prior contribution (control)			Y			Y
*N*	97,149			97,149		

This table reports the estimated relationship of contribution likelihood and contribution amount as a function of our treatment interventions during our experimental period (October 3 to December 31). We compare treatment interventions relative to a standard, control account statement. Columns (1) and (2) estimate the relationship between our treatments versus a standard, control statement on contribution likelihood. Columns (3) and (4) estimate the relationship between our treatments versus a standard account statement on contribution amount. Given that the majority of the sample made zero contributions, we use an inverse hyperbolic sine transformation on the total amount contributed during our experimental period. For ease of interpretation, we present the estimated parameters which will measure the percentage impact of receiving a treatment relative to the baseline specification. In specifications (2) and (4), we include a control for prior contribution status. Robust standard errors are used and shown in parentheses. Statistical significance is indicated by *, **, and *** for 10%, 5%, and 1% *P*-value, respectively, using a two-tailed test.

Next, we compare whether specifically framed follow-up SMS reminders improved contribution rates relative to receiving the redesigned, new account statement. As we show in Table S4 in the Supplemental Materials, only the Family Security SMS significantly improved both the proportion who made a voluntary contribution (P=0.037, two-tailed) and amount contributed (P=0.035, two-tailed). Comparing the effectiveness of the Family Security SMS relative to the other SMS interventions, we find a similar pattern. The Family Security SMS significantly improved voluntary contribution rates relative to all but one intervention at P<0.038, two-tailed (see Table S5). The Family Security SMS had a positive, though not statistically significant, effect on contribution likelihood relative to the Individual Goals SMS (β=0.00075, SE=0.00049, P=0.128, two-tailed). However, the Family Security SMS significantly improved the amount contributed relative to all other SMS interventions (two-sided *P* values <0.037). Overall, the Family Security SMS boosted contribution rates by 21.5% relative to the new statement and between 15.5% and 33.9% relative to the other SMS interventions (see Fig. S4).

While we find a significant positive effect of the Family Security SMS overall, it is possible that the magnitude or direction of the treatment effect may vary depending on the particular subpopulation. Determining whether heterogeneous treatment effects exist across demographic subgroups may inform how firms and policymakers can effectively implement interventions at scale (14, 21). Identifying if an intervention will have a stronger or weaker effect for particular subgroups can also provide insight for whether an intervention should be scaled to everyone or whether an intervention should be sent out more strategically to those who are most likely to benefit (7, 16). Uncovering whether differences exist early on is particularly important in the domain of financial planning and savings decisions, where the financial benefits of contributing can be substantial, compounding over time. Determining how to best scale interventions to the 2 million customers in the current firm and to the 50 million AFORE account holders more broadly is imperative.

Prior research has found that using recursive partitioning techniques, which use a data-driven approach to identifying heterogeneity, may be particularly advantageous for randomized controlled trials conducted in developing countries where there may be significant, cultural, behavioral, and contextual differences compared to where interventions were previously conducted (9, 14). Prior expectations of effects and where interactions may exist across subgroups may not be known. Given the policy interest in scaling successful interventions, using recursive partitioning tools to determine whether the treatment may have stronger or weaker than average treatment effects can be advantageous.

### Identifying heterogeneity using recursive partitioning

Recent work proposes that taking a data-driven approach to heterogeneity via recursive partitioning may allow researchers to better identify heterogeneity while avoiding the perils of overfitting or ad-hoc data analysis (9, 10, 14). In particular, we leverage two machine-learning algorithms, Causal Tree and Causal Forest, to determine whether heterogeneous treatment effects exist by identifying subpopulations where there may be lower-than-average or higher-than-average treatment effects. We provide an in-depth guide of how to implement both approaches in Appendix C of the Supplemental Materials.

First, we start with Causal Tree. The Causal Tree procedure builds on the classic classification and regression tree procedure of (22), with a few key differences. Instead of using the same data for training and prediction, the Causal Tree method uses an “honesty” approach, which allows one to estimate a model with no restrictions on the model complexity, and also allays concerns of overfitting. To achieve this, the data are randomly split into two samples: 50% of the sample is used as a training sample, with the other 50% being used as the test sample. Tree construction and treatment effect estimation can then be broken into two stages. In the first stage, the training sample is used to construct the tree, identifying heterogeneity across the independent attributes—i.e. age and gender. Since it is not necessary to specify the functional form of the relationship between the attributes and the outcome a priori, the training stage allows for identification of heterogeneity across flexibly defined partitions of the space of the independent variables.^a^ As we will describe further below, treatment effects are estimated on the test sample, rather than the training sample, which will help avoid problems with spurious correlation.^b^ We compare only those in the Family Security SMS treatment to those in the standard account statement group, setting the minimum number of observations in any given node to 20, and chose the tree complexity through cross-validation.

**Fig. 3.**
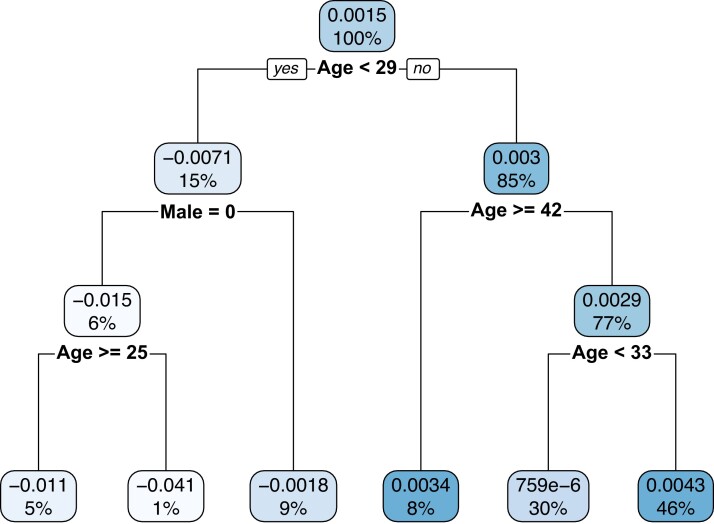
Causal tree dendrogram comparing Family Security SMS to control. This figure depicts the dendrogram produced by running Causal Tree on our training data where we compare the estimated treatment effects between the Family Security SMS intervention and the Standard Account Statement on the likelihood to make a contribution during our experimental period (October 3 to December 31). The sample is restricted to those individuals who are in the Control or the Family Security SMS conditions, with the sample size totaling to 27,755 individuals. Each bubble shows the estimated average treatment effect on the likelihood of making a contribution prior to a particular split, as well as the population prior to that split.

The results from the first stage of the Causal Tree approach are shown in Fig. 3. The top of each branch of the dendrogram represents the magnitude and direction of the Family Security SMS treatment effect for each subgroup, with each brand also indicating the proportion of the population that is within each respective subpopulation. The lines under each bubble show how the algorithm splits the data. As shown in Fig. 3, the strongest split occurs at age 29. Those who are under 29, show a stronger, but negative, treatment effect relative to those between the ages of 29 and 42, who show a positive effect. There is a weaker, but still positive effect for those over the age of 42. We also check for robustness by varying the minimum number per node from 2 to 20, and find that the partitions identified were reasonably robust across samples. For some larger values of this parameter we found that a shorter tree would result which would have splits at age 29, and then along gender below that.

In the second stage, these heterogeneous treatment effects identified using the splits in the training stage are estimated using the remaining 50% of the sample that serves as the “test” sample. Crucially, because we use an “honesty” approach, we ensure that the data used for the training stage and selecting the model is different from the data used for estimating the treatment effects during the test stage helping avoid falsely finding treatment effect heterogeneity that is driven solely by spurious correlation. Effectively, the “honesty” approach ensures that “the asymptotic properties of treatment effect estimates within the partitions are the same as if the partition had been exogenously given,” (p. 7354) (9). To put confidence bounds on the estimated treatment effects, we measure contribution likelihood as a function of the interaction of the Family Security SMS along different age and gender-based partitions defined by the dendrogram in Fig. 3. We present the estimates of the test stage in Table S6 of the Supplemental Materials.

**Fig. 4.**
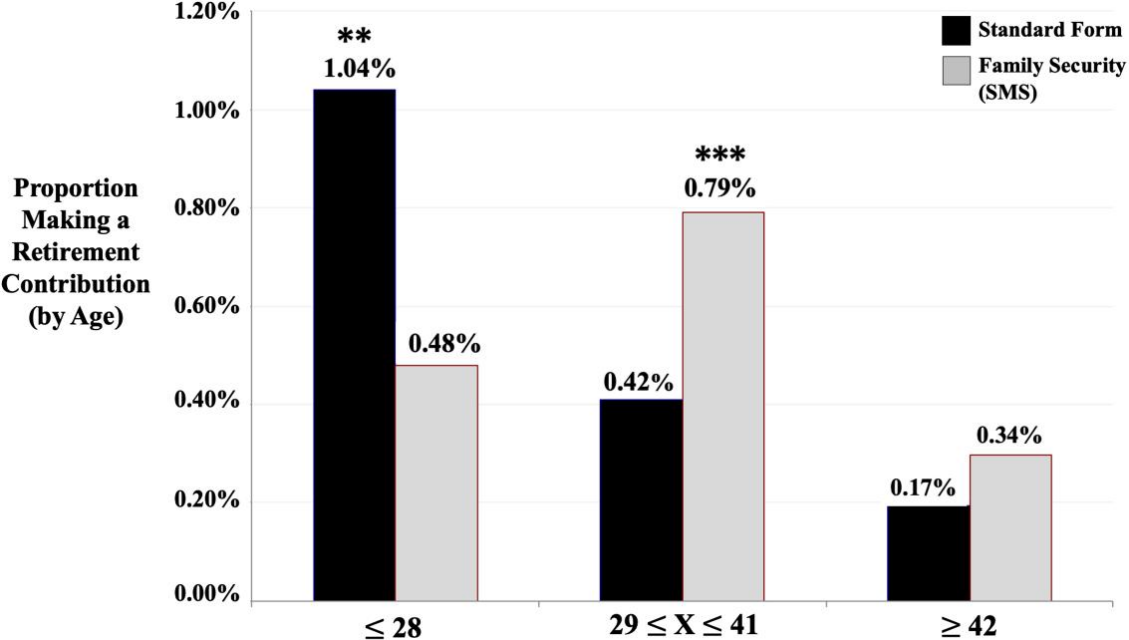
Percent making a retirement contribution, by age. This figure depicts the proportion making a voluntary retirement savings contribution as a function of receiving either the Family Security SMS intervention or the Standard Account Statement split by our three age buckets: those who are 28 and younger, those between 29 and 41, and those who are 42 and older. The sample is restricted to individuals in the control or Family Security SMS conditions with the sample size totaling to 27,755 individuals. Statistical significance is indicated by *, **, and *** for 10%, 5%, and 1% *P*-value, respectively, using a two-tailed test.

We also test the estimated treatment effects with the age splits identified using the full data via OLS and present the results in Fig. 4. Though there is an overall positive effect of the Family Security SMS, we find evidence of heterogeneous treatment effects as a function of age: Those who are 28 or younger are significantly *less* likely to make a voluntary contribution if they receive the Family Security SMS intervention relative to receiving the standard statement (β=−0.00563, Robust SE=0.00272, P=0.039, two-tailed). However, those between 29 and 41 show statistically significant positive treatment effects of the Family Security intervention (β=0.00229, Robust SE=0.00056, P<0.001, two-tailed). There is also a positive treatment effect for those 42 and older, though this is not statistically significant at the P<0.05 level, two-tailed.^c^ Using a broader age cutoff of 29 and older produces statistically significant positive treatment effects of the Family Security intervention relative to the standard account treatment (β=0.00352, Robust SE=0.00098, P<0.001, two-tailed). Finally, we show that the Family Security SMS shows significantly positive effects relative to the new statement alone and relative to the other SMS intervention for those over the age of 29 (see Tables S7 and S8 provided in the Supplemental Materials).

Despite producing unbiased estimates of heterogeneous treatment effects, one drawback of the Causal Tree procedure is that the estimates are based only on a single tree. The results using Causal Tree can also be sensitive to the subsample of the data used, meaning that prediction error can be high. It is possible that some of the partitions shown near the bottom of Fig. 3 could disappear or be replaced with other small partition if a different 50% subsample is used. While the earlier splits that occur around the ages of 29 and 42, as well as along gender lines for younger individuals, are robust to various subsamples, reducing prediction variance is critical particularly for scaling. In contrast to Causal Tree, the Causal Forest procedure constructs predictions by averaging over many trees grown on various subsamples of the data, lower prediction variance and increasing the stability of the results (10, 23, 24).

**Fig. 5.**
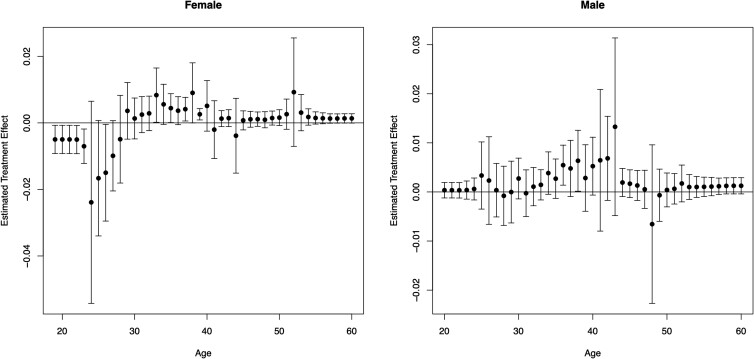
Causal forest: conditional average treatment effects by gender and age. This figure depicts the first stage plots produced by running Causal Forest across age, comparing the Family Security SMS intervention to the control group on the likelihood to make a contribution during the experimental period (October 3 to December 31). The sample is restricted to those individuals who are in the Control or the Family Security SMS conditions, with the sample size totaling to 27,755 individuals. Plots are split by gender with error bars represent 95% confidence intervals.

We identify heterogeneous treatment effect using the Generalized Random Forest approach (10), once again, comparing the voluntary contribution rates for those who received the Family Security SMS intervention relative to those who received the standard account statement. We present the results of the first “test” stage of Causal Forest in Fig. 5. We observe a negative effect of the Family Security SMS for younger individuals and a positive effect for those above the age of 29. However, the backfire effect appears to be particularly pronounced for women relative to men. Notably, we observe that the Family Security SMS improved contribution rates for those above the age of 29, regardless of gender.

The results from the “test” stage of Causal Forest also uncovered significant heterogeneity in other interventions. As we show in Table 3, we observe that all but one of the behavioral interventions produce significant backfire effects relative to the standard account statement, particularly for women at P<0.05, two-tailed. There is also a marginally significant backfire effect for Fresh Start SMS relative to the standard account statement (P=0.070, two-tailed). In fact, we find no worse effects of the Family Security SMS relative to the other interventions (see Table S8). Importantly, the Family Security SMS was the only intervention to robustly improve contribution rates for those above the age of 29, regardless of gender (P<0.05, two-tailed).^d^

**Table 3.** Estimate treatment effect by age and gender (all treatments).

*Age bracket*	New statement (No SMS)	Basic Alert SMS	Fresh Start SMS	Small Amounts SMS	Individual Goals SMS	Family Security SMS
Female						
Age ≤ 28	− 0.01150${}^{**}$	− 0.01382${}^{**}$	− 0.01041${}^{*}$	− 0.01244${}^{**}$	− 0.01700${}^{**}$	− 0.01456${}^{**}$
	(0.00566)	(0.00547)	(0.00588)	(0.00561)	(0.00512)	(0.00536)
29 ≤ Age ≤ 41	0.00254	0.00165	0.00353${}^{**}$	0.00230	0.00077	0.00529${}^{**}$
	(0.0016)	(0.00153)	(0.00168)	(0.00158)	(0.00147)	(0.00179)
Age ≥ 42	0.00764${}^{*}$	0.0000	0.0000	0.0000	0.00155	0.0000
	(0.00402)	(0.00211)	(0.0022)	(0.00215)	(0.00262)	(0.00219)
Male						
Age ≤ 28	0.0000	0.00242	0.00218	0.00368	0.00227	0.00066
	(0.00265)	(0.00301)	(0.00293)	(0.00312)	(0.00294)	(0.00269)
29 ≤ Age ≤ 41	0.00005	0.00232${}^{*}$	0.00107	− 0.00074	0.00044	0.0026${}^{**}$
	(0.00118)	(0.0013)	(0.0012)	(0.00109)	(0.00117)	(0.00131)
Age ≥ 42	− 0.00204	0.0019	− 0.0020	− 0.00193	0.00174	0.00349
	(0.00203)	(0.00333)	(0.0020)	(0.00194)	(0.00335)	(0.00376)

This table reports the estimated treatment effects and confidence intervals derived from generalized random forest estimation, comparing all intervention to the control group on the likelihood to make a contribution during the experimental period. This table presents the estimates that were derived by using a supplied procedure that estimates the conditional average treatment effect for subsets of the data. Positive coefficients represent the behavioral intervention improving contributions relative to the standard account statement, while negative coefficients represent the behavioral intervention decreasing contributions. Robust standard errors are used and shown in parentheses. Statistical significance is indicated by *, **, and *** for 10%, 5%, and 1% *P*-value, respectively, using a two-tailed test. All regressions include a constant and controls for prior contribution status.

The heterogeneous treatment effects across age and gender produce significantly lower-than-average and higher-than-average treatment effects relative to the estimates of each of the interventions overall (see Tables S9 and S10). For women, we observe a larger and positive treatment effect for the Family Security SMS for those in the middle age bin (29 to 41) relative to the ATE of the Family Security SMS. For women who are 28 and younger, we observe statistically significant backfire effects for all but one of the interventions, relative to the average treatment effect (two-tailed unadjusted P<0.05). For men, on the other hand, we have insufficient evidence to reject the null hypotheses that the HTE is significantly different than the ATE (two-tailed unadjusted P<0.10). Once again, only the Family Security SMS produces positive effects for those above the age of 29, regardless of gender (two-tailed unadjusted P<0.05).

It is worth noting that it may be common for researchers to rely on OLS with interaction terms for estimating heterogeneous treatment effects. OLS approaches for identifying heterogeneity are typically used in cases where researchers have prior expectation of effects and where a preanalysis plan is specified. In these instances, the functional form of the relationship between the treatment effects and the attributes of the additional covariates (e.g. age, gender) is known. In our setting, this is not the case. To illustrate the potential limitations of OLS for identifying heterogeneity in treatment effects when researchers have no prior expectations, we analyzed our data with OLS including interactions between our Family Security SMS, gender, and different age specifications (i.e. linear-age trend, quadratic-trend, 10-year and 5-year age bins). We present these results in Tables S18 and S19 of the Supplemental Materials. The results show that we are unable to accurately identify heterogeneity. However, if the correct age cutpoints are known in advance or prespecified in the analysis—e.g. if a researcher predicted the age and gender splits ahead of time—then OLS performs similarly to the Causal Tree and Causal Forest models identifying both the backfire of all interventions for younger women and the positive effects of the Family Security SMS for those above 29 (see Table S20). Our results highlight the benefit of leveraging data-driven approaches, such as Causal Tree and Causal Forest, to identify heterogeneous treatment effects, particularly in contexts where (i) researchers may not have prespecified expectations of effects and (ii) if the goal is to make out-of-sample predictions and scale interventions to broader populations (11, 25, 26).

To gain some potential insight for why the precise age breakpoints may produce heterogeneous treatment effects for the Family Security treatment, we turned to Census data in Mexico on family and labor trends. According to the Census, the average age for a first marriage for women was 26 and 26.5 for men, during the time of our experiment. Though we do not have data indicating whether people have children, the direction of the treatment effect at age 29 is consistent with these trends. Relative to the standard account statement, it is possible that receiving a Family Security SMS may prompt people to worry less about retirement savings until after having children or may even shift younger people to think more about their parents’ retirement. In both scenarios, the same intervention may significantly lower contributions for their own retirement, but may significantly boost contributions when individuals are a bit older. While these explanations are speculative and do not causally determine the precise mechanism driving our heterogeneous treatment effects, future research specifically testing the potential psychological mechanisms underlying this heterogeneity, in this cultural setting and more broadly, are imperative.

Taken together, we find that the Family Security SMS produces heterogeneous effects, particularly for women, significantly decreasing voluntary contributions for those below the age of 29 but significantly increasing contributions for those 29 and older. For men, the Family Security SMS does not affect contribution rates for those under the age of 29. Instead, it only produces a significantly positive effect, increasing contributions for those above the age of 31 (P<0.05 level, two-tailed). Notably, though we observe a significant backfire for those under 29 relative to the standard account statement, we do not find sufficient evidence that the Family Security SMS performs significantly worse relative to other behavioral interventions. Future research should explore (i) what mechanisms are driving younger people, and younger women in particular, to respond negatively to the behavioral interventions used in our setting, and (ii) whether other types of behavioral interventions may better motivate younger people to save for retirement.

### Measuring post-intervention persistence

One natural question is whether the effectiveness of the Family Security SMS intervention improves voluntary savings contributions persists beyond the experimental period or if the effects are short-lived. For researchers and policymakers, testing for persistence is particularly useful for providing potential insights into what behavioral factors may contribute to why people fail to save for retirement. If the failure to save for retirement is due to the perception that retirement savings only benefit the individual, then sending a Family Security SMS may shift this perception and have a lasting impact post-intervention. For policymakers and practitioners, determining whether the upfront costs of sending the initial SMS can endure beyond the experimental period or whether follow-up SMS reminders may be needed can provide more accurate cost estimates moving forward.

To test for persistence, we obtained contribution data two months after our experiment had concluded, which was roughly five months after the initial interventions were sent. Regardless of the original experimental condition, all individuals returned back to the receiving the standard account statement at the beginning of the new year. In addition, no additional SMS messages were sent following the original intervention message in October. Using the same ANCOVA specifications from our initial analyses, we tested whether the various behavioral interventions sent in October influenced contribution rates in the two-month period (January 1 to February 28) post-experiment.

Table 4 presents the estimates of our intervention groups relative to the standard account statement, baseline group on contribution likelihood and total contribution amount in the two-month period post-experiment. Those who received the Family Security SMS in October were significantly more likely to make a voluntary retirement contribution and contributed more money to their retirement account, in the two-month period following our experiment ($P^{\prime }s<0.05$, two-tailed).^e^ Though the magnitude of the effect is weaker, a beta-coefficient comparison of the family effect from our experimental period and the post-treatment time period shows a 77% persistence rate.

**Table 4.** Measuring persistence: Contribution likelihood and amount contributed post-experiment.

Dependent variable:	Contribution likelihood			Contribution amount ($) *(IHS transformed)*		
*Condition*	Mean	(1)	(2)	Mean	(3)	(4)
Standard account statement *(Baseline)*	0.49%	–	–	$93.58	–	–
New statement (No SMS)	0.54%	0.00059	0.00058	$123.27	0.00680	0.00672${}^{*}$
		(0.00086)	(0.00047)		(0.00726)	(0.00404)
New statement + Basic Alert SMS	0.55%	0.00059	0.00014	$120.43	0.00640	0.00262
		(0.00088)	(0.00048)		(0.00723)	(0.00446)
New statement + Fresh Start SMS	0.58%	0.00094	0.00056	$95.76	0.00780	0.00455
		(0.00088)	(0.00051)		(0.00724)	(0.00415)
New statement + Small Amounts SMS	0.49%	0.00001	0.00016	$72.76	− 0.00061	0.00069
		(0.00084)	(0.00052)		0.00686)	(0.00435)
New statement + Individual Goals SMS	0.44%	− 0.00043	0.0006	$129.78	− 0.00356	0.00510
		(0.00082)	(0.0005)		(0.00674)	(0.00413)
New statement + Family Security	0.66%	0.00168${}^{*}$	0.00117${}^{***}$	$138.89	0.01503${}^{**}$	0.01078${}^{**}$
		(0.00091)	(0.00053)		(0.00757)	(0.00444)
Prior contribution (control variable)			Y			Y
*N*	97,149			97,149		

This table reports the estimated relationship of contribution likelihood and contribution amount as a function of our treatment interventions during the two-month period *following* our experimental period and after all individuals went back to the standard account statement. Columns (1) and (2) estimate the relationship between our treatments interventions versus a standard account statement on contribution likelihood. Columns (3) and (4) estimate the relationship between our treatments interventions versus the standard account statement on total contribution amount in the two-month period following the conclusion of the experiment using an inverse hyperbolic sine transformation. Columns (2) and (4) include a control for prior contribution status. All regressions include a constant. Robust standard errors are used and shown in parentheses. Statistical significance is indicated by *, **, and *** for 10%, 5%, and 1% *P*-value, respectively, using a two-tailed test.

In addition, we observe heterogeneous treatment effects based on age. We find that those who are above the age of 29 and who received the Family Security SMS in the prior period are more likely to make a contribution relative to those who received the standard account statement or any of the other behavioral interventions (P<0.05, two-tailed, see Tables S22–S24 of the Supplemental Materials). Promisingly, we observe no evidence that the Family Security SMS has persistent backfire effects relative to the standard account statement or any of the other behavioral intervention (see Tables S23 and S24). These findings suggest that the beneficial effects of the Family Security SMS may persist longer than the maleficent effects.

### Implications for scaling

Identifying heterogeneous treatment effects has important implications for policy and firm decision making. Scaling the Family Security SMS based on OLS results where only an average treatment effect is identified would imply using an one-size-fits-all approach, sending the Family Security SMS to everyone in the population. However, doing so could lower the number of contributors, total contribution amounts, and may also decrease firm profits. Instead, using a more strategic, segmented approach that accounts for treatment heterogeneity by sending the Family Security SMS only to those who respond positively to the intervention—i.e. those who are 29 and above—and sending those who are 28 and below the status quo, standard account statement without follow-up SMS reminders may significantly boost effectiveness.

We quantify the impact of scaling the Family Security SMS using either a one-size-fits-all approach or using a strategic, more segmented approach by performing a counterfactual exercise. We assume that the firm has a customer base of two million individuals and that this customer base matches our sample demographically. This is a reasonable assumption given that the firm had roughly two million customers at the time of our experiment and ensured that we used a representative sample that matched their customer base at the time. We take the proportion of individuals in each age bracket indicated in the Causal Tree results from our experimental sample to be reflective of the firm’s overall sample—14.76% in the 19–28 age group, 76.68% in the 29–41 age bracket and 9.56% in the 42 and older age bracket. We then compute an estimate of the effect of the Family Security SMS on the number of predicted contributors (and overall contributions) in each age bracket by computing the raw probability of contribution (average amount contributed) for the Family Security SMS and the standard account statement, multiplying this probability by the overall number of individuals in each age bracket, and then taking the difference.^f^ We compute a counterfactual estimate of the impact of the one-size-fits all approach by simply adding up the predicted number of contributors (or contributions) across the three brackets, while for the more targeted approach we only add the effects from the two age brackets containing above 29 years and older. In other words, we assume the firm does not target individuals under 29, since their response to the intervention is negative.^g^

Using this counterfactual exercise, we find that sending the Family Security SMS using a one-size-fits-all approach would result in 4,341 new contributors per period relative to the status quo, improving the number of contributors by 44%. In contrast, a strategic, more segmented approach would result in 6,004 new contributors per period, improving the number of contributors by 61% relative to the status quo and 38% relative to a one-size-fits-all approach. The total amount of money contributed would also increase in both scenarios, though more so when using a segmented approach. Sending a standard account statement is estimated to increase contributions by roughly $229 million pesos (∼$10,750,00 USD) in total contributions per period. Scaling the Family Security SMS using a segmented approach will increase contributions by $380 million pesos (∼$17,860,00 USD), relative to a $364 million pesos (∼$17,100,00 USD) increase using a one-size-fits-all approach. While it is difficult to estimate the precise amount that this would net for contributors at the time of retirement, it is plausible that the difference could be sizable at the time of retirement. The average age of the firm’s customer base is relatively young at 33.8 years. The annual rate while the annual rate of return for the firm has ranged from a low of 2.5% to 15.5% over the last five years. If the Family Security SMS motivated a 33 year-old person to start making regular contributions of $250 MXN contribution (∼$12.85 USD) per quarter, this could result in a difference of between $20,000 MXN (interest rate of 2.5%) and $730,000 MXN (interest rate of 15%) when the person is 65 and at the time of retirement.

Finally, firm profitability is also improved if a more strategic, segmented approach is implemented. To calculate firm profitability, we need to make some additional assumptions. First, we assume that the firm’s increase in profits from an additional peso invested is equal to the fees the firm would collect from that investment. This assumption is line with prior literature (27), where it is assumed that the marginal cost of taking on additional contributions is zero. Additionally, consistent with their research we assume that the firm’s fee income is a fixed percentage of contributions. Because we cannot identify the firm, we assume that the fee income received by the firm is 20% of contributions, which is in line with the overall market rate.^h^ We evaluate the profitability of both the one-size-fits-all approach versus the strategic, segmented approach by taking the fee revenues minus the cost of sending the two SMS reminder messages ($2.20 MXN per text, $4.4 MXN total or $0.10 USD total) either to the entire two million customers or only to those above the age of 29, which is roughly 1,704,800 individuals.

Scaling up our intervention using a one-size-fits-all approach would increase profits by $18,097,700 pesos ($850,600 USD). In comparison, a more strategic, segmented approach that accounts for heterogeneous treatment effects would increase profits by $22,581,000 pesos ($1,061,300 USD). Although firm profitability will increase either approach is used, targeting the Family Security SMS to the segments that respond positively based on HTE is significantly more profitable. Notably, these estimates may be conservative as they are based on the estimates from just one period. People who start making voluntary contributions are very likely to do so in the future, potentially amplifying the positive effects post-intervention. We observe that 77% of those who contributed in the Family Security SMS intervention contributed in the following period. This estimate is similarly to the 81% repeat contribution rates that we observe in our overall sample. Policymakers, firms, and citizens may all benefit from identifying heterogeneity and scaling interventions in a more tailored and targeted manner.

## Discussion

In this paper, we use a large-scale field experiment (N=97,149) and show that framing retirement savings as a way to benefit one’s family can significantly increase contribution rates relative to a standard account statement or other common behavioral interventions. Leveraging recursive partitioning techniques, we identify that the overall positive treatment effect masks subpopulations where the treatment is even more effective and other groups where the treatment has a significant negative effect, decreasing contribution rates.

Our findings have important implications for behavioral research and policy. Behavioral science research has predominately focused on estimating average treatment effects and conditional average treatment effects to identify treatment heterogeneity. However, there are significant cultural and behavioral factors in Mexico that may limit the ability to make clear predictions for which subgroups may produce lower or higher than average treatment effects. In these instances, data-driven approaches can aid in identifying heterogeneity, particularly in cases where there is interest in scaling interventions beyond the experimental sample. While there is recent evidence that behavioral interventions may have attenuated effects when interventions are scaled broadly (6, 7), it is quite possible that identifying heterogeneous treatment effects may be essential. The effectiveness of many behavioral interventions in behavioral economics, choice architecture, and behavioral science may in fact be undervalued if not heterogeneously applied—both in terms of theory development and policy execution.

In our setting, identifying heterogeneity is crucial. Very small differences in contribution rates can add up when an intervention is scaled to a larger population. If the intervention were to be scaled up to the roughly 50 million active retirement account holders (29), then uniformly applying a behavioral intervention can produce suboptimal results compared to a more strategic and targeted approach where the Family Security SMS is sent only to subgroups who respond positively. In our setting, identifying a 0.1% improvement or decline in contribution rates could have an impact on 49,890 individuals if the intervention where to be scaled up to all active pension holders in Mexico.

Finally, it is important to acknowledge that there are limitations in our findings. Our results are only from one firm and in one cultural setting. Barriers to saving may differ in other cultural settings or even for different subgroups and who may have different constraints or structural obstacles that may hinder. It is also important to recognize that varying the simplicity of forms or sending SMS reminders will not solve the retirement savings gap alone. However, from a policy standpoint, interventions that are easy and low-cost have the potential to be adopted more readily and face fewer regulatory challenges relative to other options (e.g. increasing the mandatory contribution rate, one-on-one financial counseling or educational programs). Our results shed light on ways to maximize the effectiveness of behavioral interventions using data-driven approaches in ways that can improve financial savings behavior at scale.

## Materials and methods

### Background

Since 1997, all full-time workers in Mexico have been required to select and contribute 6.5% of their salary to one of several government-designated retirement fund administrators known as AFOREs (Administradoras de Fondos para el Retiro). Our field experiment was conducted in collaboration with one of these AFOREs. In addition to an individual’s mandatory contribution, the government contributes another 5.5% of the indexed minimum salary on the behalf of each worker. The combined total of the mandatory employee contributions, government contributions, and the accrued interest equates to employees receiving roughly 30% of their average earned salary at the time of retirement. This amount is substantially less than the target of 70% that is typically recommended by financial experts (30, 31). To close the gap, account holders are encouraged to make additional *voluntary* contributions. Voluntary contributions could be made with any frequency or amount exceeding $50 MXN ($2.35 USD). However, less than 1% of account holders choose to do so (8).

To better identify what barriers might be preventing individuals from contributing to their retirement, and to help inform our experimental design, we first conducted interviews surveying over one hundred account holders across three major cities in Mexico. We asked individuals whether they made voluntary retirement savings contributions, and if not, what prevented them from doing so. Many of the obstacles listed were driven by cognitive errors, procedural hassles, inattention, and present bias/myopia. A number of individuals did not correctly calculate how savings could grow exponentially over time or how interest rate differences across savings products could affect the amount of savings accrued (e.g. during interviews respondents stated “I cannot see how saving this small amount would actually help me in the future”; “Does it really make a difference whether I contribute my money to my bank’s savings account or my retirement fund?”) (32). With regards to procedural hassles and inattention, a number of individuals were unclear how to make a voluntary contribution and/or simply forgot to make a contribution (e.g. “I do not remember where I can even go to make a contribution or what information I would need?” and “I want to contribute but I always seem to forget”) (33, 34). Consistent with present bias and myopia, many respondents reported that the consequences of the future felt far away and abstract, particularly relative to trading off consumption in the present (35, 36). Given that the reasons stated were similar to barriers documented in past work, it is reasonable to believe that previously successful interventions conducted would be equally effective in this context. We chose five previously successful behavioral interventions aimed at improving retirement savings: simplified account statements (34), basic reminder alerts to save (37), using a pennies-a-day/small amount framing for savings (38), a fresh start appeal (39), and a concrete visualization of one’s individual future (40, 41).

Many interviewees also reported not saving because they wanted to have money easily accessible for their family. A few respondents noted that saving for their own individual retirement meant not having money available to take care of their family’s immediate needs. Two interviewees noted that they would prefer to spend money on their children rather than to save for their own retirement. A number of interviewees further noted that they were willing to sacrifice their own retirement savings to ensure that their family’s needs are met. These responses were consistent with individuals prioritizing collective welfare and interdependent goals. However, these responses also suggested that saving for retirement was seen as more of an individual benefit, rather than one that could be beneficial to one’s family or to serve a more collective goal. Alongside previously effective interventions, we also tested an undocumented intervention in the domain of financial savings—framing the decision to save for retirement as one that will secure one’s family’s financial future.

### Materials and procedure

Customers are mailed account statements every four months. Account statements contain information on how much the individual has contributed (both mandatory and voluntary), how much they expect to earn post retirement at their current savings rate, and how to make a voluntary retirement contribution. In addition, the firm had the capability to send SMS to their account holders’ mobile phones. These texts could be easily personalized and were relatively inexpensive to send to accountholders with each SMS costing $2.2 MXN ($0.10 USD). The firm had not yet used SMS prior to the start of the experiment.

In the last week of September 2016, the AFORE began mailing out the last account statement for the year. Our field experiment commenced when individuals receiving their account statement at the beginning of October and concluded at the end of the account statement period prior to account statements being sent out for the following period (December 31st). Customers were randomized into either the baseline, control group or one of six intervention groups varying the content of the account statements and/or the text messages designed to encourage voluntary retirement savings contribution.

Individuals received either the typical standard account statement (baseline, control group, see Fig. S2), a new redesigned and simplified account statement, or the new redesigned statement plus one of five SMS reminders designed to encourage savings contributions (see Fig. S3). The redesigned account statement improved the readability and visual appeal of the statement. It included a thermometer on the top-left emphasizing how well the individual was doing at saving for retirement (an indicator in the red region, which all participants received, suggested savings were inadequate) as well as information in the bottom of the form describing how to make a voluntary contribution. In addition to the new account statement, we randomized whether certain people receive one of five SMS reminders based on previously successful interventions—i.e. basic reminder alerts, pennies-a-day/small amount framing, fresh start appeals, concrete visualization of one’s individual future. As mentioned earlier, we also tested a previously undocumented intervention in the domain of financial savings, framing the decision to save for retirement as one that will secure one’s family’s financial future.

Because all treatment conditions received the same, simplified account statement, the new statement provides another useful benchmark for comparison allowing us to determine whether using a simplified statement improved contribution rates or whether specifically framed follow-up reminders were necessary. A timeline charting when customers received various interventions and when outcome variables were collected is shown in Fig. S1 of the Supplemental Materials. The stimuli used for all account statements and text messages, in English and Spanish, are also provided in Figs. S2 and S3 and Tables S1 and S2, respectively.

Our dependent variables of interest were whether individuals made a voluntary retirement contribution (coded as 1 = Yes, 0 = No) and the total contribution amount during the experimental period (i.e. prior to the next account statement being sent). Contribution amount was inverse hyperbolic sine transformed as the data was right skewed. We measured the effectiveness of each intervention relative to the standard account statement and also in comparison to each of the other treatments. All of the outcome measures that we collected are reported in the results.

Participants were unaware that they were part of a randomized experiment, and were unaware that any changes to the account statement or SMS texts were part of a randomized experiment at any point prior, during, or following the experiment period, though no deception was involved.

This research was granted a waiver of consent and conducted in conjunction with a federally approved retirement fund administrator (AFORE) that was authorized to manage individual retirement accounts and was overseen and approved by the Comisión Nacional del Sistema de Ahorro para el Retiro (CONSAR), Mexico’s national commission of the retirement savings system. No identifying information about study participants was shared with the researchers.

## Supplementary Material

pgad058_Supplementary_Data

## Data Availability

Our experiments and analysis involve confidential financial data from Mexico, and thus they cannot be released publicly. We can arrange for individuals to work with the raw data for the purpose of replication on a secure computer after arranging for a nondisclosure agreement. The research team will facilitate this process for replication. We can also arrange for individuals to receive an anonymized dataset that de-identifies any individual information.

## Footnotes

a Later in this section, we will describe some pitfalls of standard approaches, which assume a known functional relationship between treatment effect heterogeneity and independent attributes.

b For example, a standard regression tree would choose to split the space of independent variables where the correlation between the independent variables and the dependent variables are strongest. This is problematic if there are some partitions of the space of the independent variables that may produce spurious correlation between treatment effects and particular independent variables by random chance.

c We also ran Causal Tree on the other treatments, but for most of the treatments the procedure did not produce a dendrogram with any heterogeneity. One exception was the Small Amounts SMS treatment, which produced a split at ages 30 and 41. This dendrogram suggested the treatment effects are negative for individuals below 30 and positive above that.

d For a detailed breakdown of the results of each intervention using Causal Forest, please refer to Tables S11–S16 provided in the Supplemental Materials. we also present the results of several simulation studies and demonstrate that the estimated effects are robust to the number of trees and the tuning parameter values in Table S17. For a dataset of our size, we show that the Causal Forest procedure is able to recover the underlying heterogeneous treatment effects using a similar data generating process for estimated heterogeneous treatment effects, supporting the validity of the method in our application.

e The post-experiment follow-up time period is one month (31 days) shorter than the main experimental period. To compare the estimates using the equivalent time window, we also re-run our main analyses by testing whether our interventions increased the likelihood of making a voluntary savings contribution relative to the standard baseline in the 58 days period following when the new account statement was sent out (October 3) until November 30. Consistent with the main analyses, our results show a positive treatment effect for the family security SMS both in the abbreviated experimental period and the two-month follow-up period. These results can be found in Table S21 of the Supplemental Materials

f For example, suppose that in the 42 and older age bracket, the number of individuals in the standard account statement, control condition and the family SMS condition are $N_{Control}$ and $N_{Family\;SMS}$, respectively, and the number of contributors are $NC_{Control}$ and $NC_{Family\;SMS}$. Then our estimate of the impact of the family security SMS intervention on the number of contributors for this subgroup would be $2,000,000 × 0.0956 × ($NC_{Family\;SMS}/N_{Family\;SMS})-(NC_{Control}/N_{Control}$). For contribution amounts, we perform a similar calculation, except we replace $NC_{Control}$ and $NC_{Family\;SMS}$ with the total amount contributed in each condition for the relevant subgroup.

g An alternative approach to the one we use, which would rely on the Causal Forest estimates, would compute the change in contribution probability for each value of age and gender, and in the case of targeting would only apply where the change was predicted to be positive by Causal Forest. We do not take this approach because we found that many of the CATEs for specific values of age and gender were not precisely estimated. However, the CATEs suggested by the Causal Tree estimates were statistically significant.

h Hastings and Hortaçsu (27) calculate that one year after the government’s initial reform, 23% of an individual’s contribution would be paid as fees to the firm. Their calculation relied on computing average load fees (the amount charged when money is transferred to someone’s account). At that time, firms charged both load fees (on transfers) and balance fees (on balances held). However, the government has since reformed the pension system. Firms today only charge balance fees. In 2012, after the pension system reform, median losses in wealth from firm balance fees were 19.9% (28).
