# Correction: Identifying heterogeneity using recursive partitioning: evidence from SMS nudges encouraging voluntary retirement savings in Mexico

**DOI:** 10.1093/pnasnexus/pgad449

**Published:** 2023-12-27

This is a correction to: Avni M Shah, Matthew Osborne, Jaclyn Lefkowitz Kalter, Andrew Fertig, Alissa Fishbane, Dilip Soman, Identifying heterogeneity using recursive partitioning: evidence from SMS nudges encouraging voluntary retirement savings in Mexico, *PNAS Nexus*, Volume 2, Issue 5, May 2023, pgad058, https://doi.org/10.1093/pnasnexus/pgad058

In the originally published version of this manuscript there was a tyhpographiocal error in the third author's name. This should read: “Jaclyn Lefkowitz Kalter” instead of: “Jacyln Lefkowitz Kalter”.

The error has been emended in the article.

