# Supplementary material for: Identifying heterogeneity using recursive partitioning: evidence from SMS nudges encouraging voluntary retirement savings in Mexico
Source: PNAS Nexus. 2023 Mar 1;2(5):pgad058. doi: 10.1093/pnasnexus/pgad058 (PMC10156144; doi:10.1093/pnasnexus/pgad058)
Supplement: pgad058_Supplementary_Data [file pgad058_supplementary_data.pdf]

!

Supplemental Appendix

This is the Supplemental Appendix for “Identifying Data-Driven Heterogeneity Using Recursive Partitioning: Evidence from SMS Nudges Encouraging Voluntary Retirement Savings in Mexico"

- Appendix A contains information on the timeline of the experiment and contains samples of the stimuli used for the Field Experiment including both original Spanish and English translations.
- Appendix B tests the robustness of the Family Security SMS results. This section includes robustness tests for contribution amount using log transformation and estimates the treatment effects using the New Statement and Family Security SMS as baseline.
- Appendix C provides an in-depth guide to using recursive partitioning approaches for both Causal Tree and Causal Forest.
- Appendix D presents the results of the second stage of Causal Tree, estimating the splits implied from the first stage using the 50% holdout sample.
- Appendix E provides the results comparing the heterogeneous treatment effects relative to the average treatment effects.
- Appendix F provides the plots from the first stage of the Causal Forest exercise, as well as the estimates of the treatment effects produced via Causal Forest for all behavioral interventions.
- Appendix G contains details of the simulation study used for the Causal Forest approach.
- Appendix H shows the results for identifying heterogeneous treatment effects using OLS.
- Appendix I presents a detailed analysis of the persistence results post-intervention, using different time intervals and age specifications.

A Timeline of Experiment and Stimuli Used

Figure S1: Visualization of Intervention and Outcome Measurement Timeline

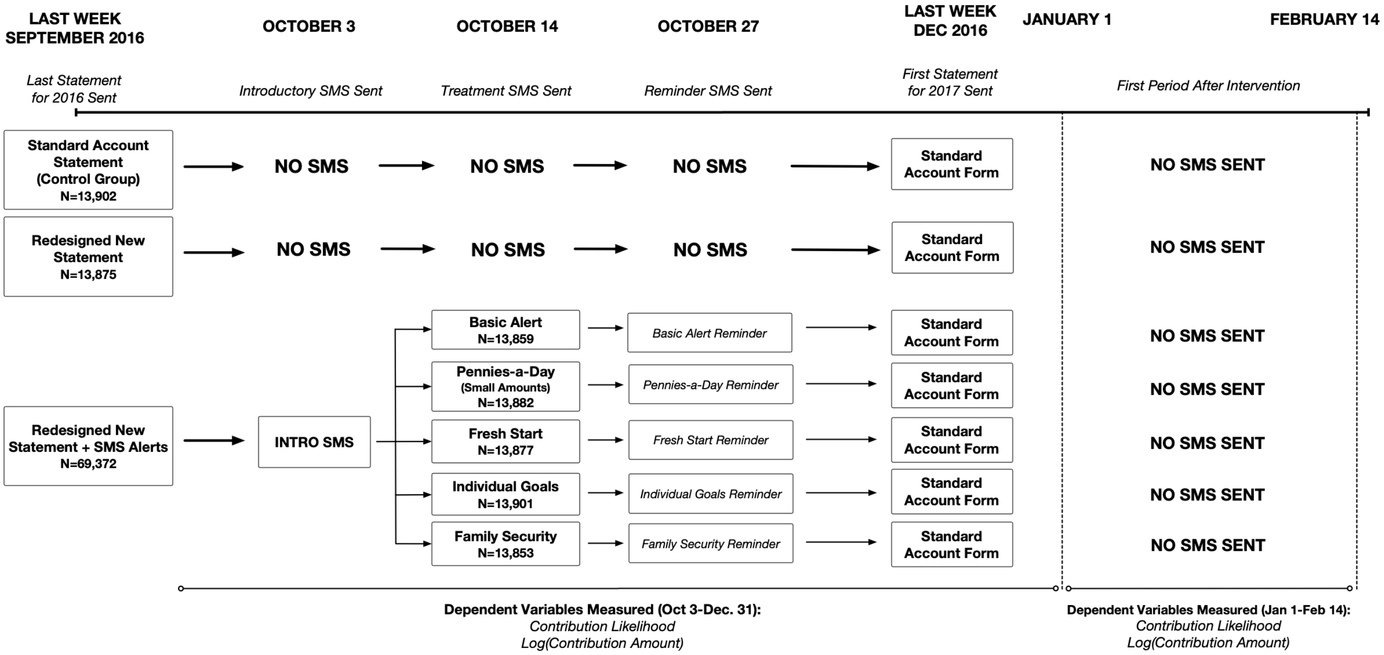

Figure S2: Standard Account Statement (Control Group)

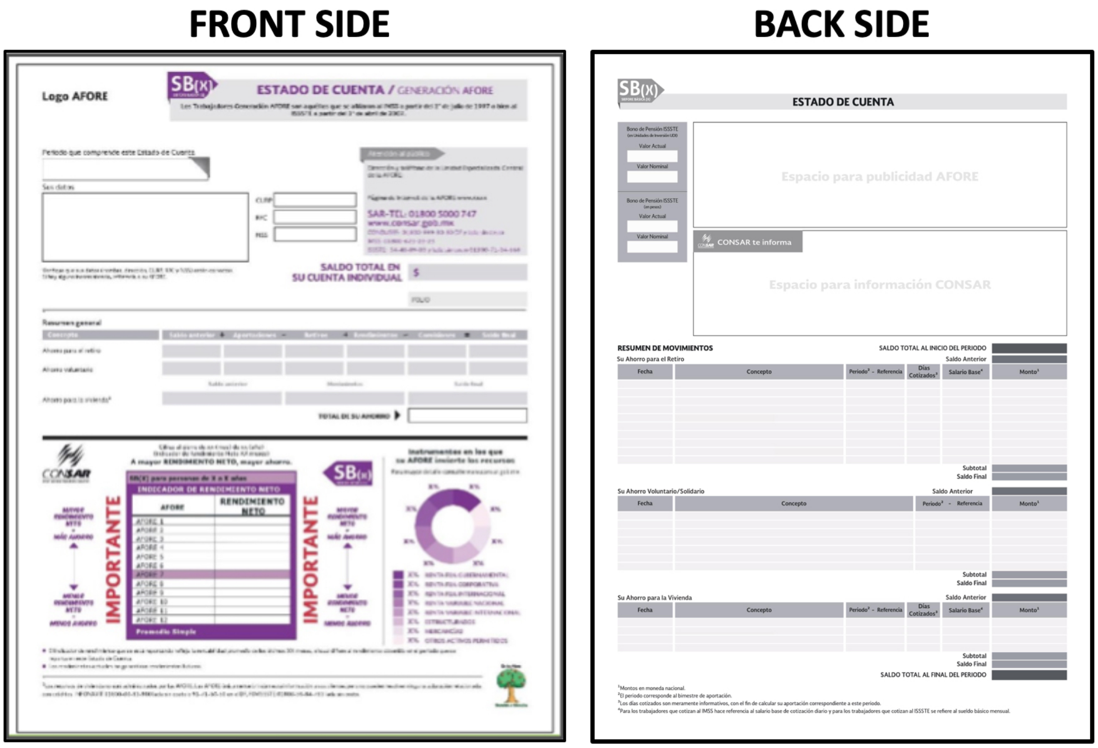

Figure S3: New Account Statement (All Treatment Groups)

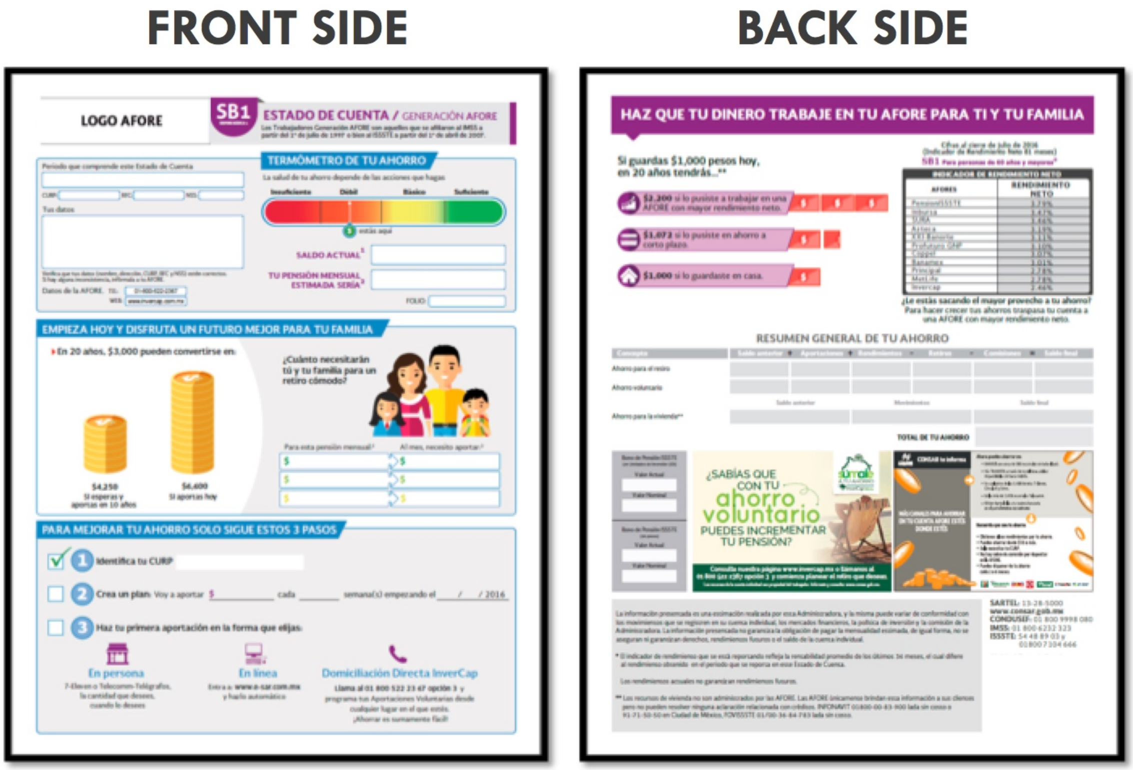

Table S1: Description of Treatments (English Translation)

Condition	Sent Introductory Text? (October 3)	Introductory Message	Sent Treatment Text? (October 14)	First Message	Reminder Message (October 27)
Control	No	No	No		No
New Statement	No	No	No		No
New Statement + Basic Alert	Yes	Hi [[Name]], Look out for tips from your Afore through SMS to remind you about your Voluntary Savings. These messages will help you improve your future.	Yes	Hi [[Name]], check out the tips in your Afore account statement. Improve your future: Contribute to your Afore Account at a 7Eleven or Telecomm with your CURP or call 018005222367 option 3	Hi [[Name]], Improve your future TODAY! Contribute to your Afore Account at a 7Eleven or Telecomm with your CURP or call 018005222367 option 3
New Statement + Fresh Start	Yes	Hi [[Name]], Look out for tips from your Afore through SMS to remind you about your Voluntary Savings. These messages will help you improve your future.	Yes	Hi [[Name]], check out the tips in your Afore account statement. Start next week off on the right foot and take the first step to save for your future. Add to your Voluntary Savings account and improve your future: Contribute to your Afore Account at a 7Eleven or Telecomm with your CURP or call 018005222367 option 3	Hi [[Name]], it's almost a new month, start your savings NOW: Contribute to your Afore Account at a 7Eleven or Telecomm with your CURP or call 018005222367 option 3
New Statement + Small Amounts	Yes	Hi [[Name]], Look out for tips from your Afore through SMS to remind you about your Voluntary Savings. These messages will help you improve your future.	Yes	Hi [[Name]], check out the tips in your Afore account statement. Little by little goes a long way: Improve your future by setting aside the cost of a small coffee per day. Start with $10 pesos a day and obtain large benefits in the future: Contribute to your Afore Account at a 7Eleven or Telecomm with your CURP or call 018005222367 option 3	Hi [[Name]], remember that small amounts improve your future: Contribute to your Afore Account at a 7Eleven or Telecomm with your CURP or call 018005222367 option 3
New Statement + Individual Goals	Yes	Hi [[Name]], Look out for tips from your Afore through SMS to remind you about your Voluntary Savings. These messages will help you improve your future.	Yes	Hi [[Name]], check out the tips in your Afore account statement. Set your goals TODAY. Plan for your future. Improve your future and reach your goals TODAY with Voluntary Savings: Contribute to your Afore Account at a 7Eleven or Telecomm with your CURP or call 018005222367 option 3	Hi [[Name]], setting a goal is the first step to change! Contribute to your Afore Account at a 7Eleven or Telecomm with your CURP or call 018005222367 option 3
New Statement + Family Security	Yes	Hi [[Name]], Look out for tips from your Afore through SMS to remind you about your Voluntary Savings. These messages will help you improve your future.	Yes	Hi [[Name]], check out the tips in your Afore account statement. Act TODAY to improve your family's future. Save for the expenses that will come. Secure your future and that of your family TODAY with Voluntary Savings: Contribute to your Afore Account at a 7Eleven or Telecomm with your CURP or call 018005222367 option 3	Hi [[Name]], it's never too late to secure a better future for you and your family: Contribute to your Afore Account at a 7Eleven or Telecomm with your CURP or call 018005222367 option 3

Table S2: Description of Treatments (Spanish)

Condition	Sent Introductory Text? (October 3)	Introductory Message	Sent Treatment Text? (October 14)	First Message	Reminder Message (October 27)
Control	No	No	No		No
New Statement	No	No	No		No
New Statement + Basic Alert	Yes	Hola [[Nombre]], ¡Espera tips de InverCap Afore via SMS para recordarte de tu Ahorro Voluntario! Estos mensajes te ayudaran a mejorar tu futuro.	Yes	Hola [[Nombre]], viste los nuevos tips en tu estado de cuenta Afore? Mejora tu futuro: Aporta en 7Eleven o Telecomm con tu CURP o llama al 018005222367 ext3	Hola [[Nombre]], mejora tu futuro ¡HOY! Aporta a tu cuenta InverCap en 7Eleven o Telecomm con tu CURP o llama al 018005222367 opcion 3
New Statement + Fresh Start	Yes	Hola [[Nombre]], ¡Espera tips de InverCap Afore via SMS para recordarte de tu Ahorro Voluntario! Estos mensajes te ayudaran a mejorar tu futuro.	Yes	Hola [[Nombre]], viste los tips en tu estado de cuenta Afore? Empieza la proxima semana con el pie derecho y toma el primer paso para guardar para tu futuro. Siguiente mensaje de acción (después de unos segundos): # Sumale a tu cuenta de Ahorro Voluntario y mejora tu futuro: Aporta a tu cuenta Afore en 7Eleven o Telecomm con tu CURP o llama al 018005222367 opcion 3	Hola [[Nombre]], un nuevo mes, otro momento para iniciar tu ahorro: Aporta a tu cuenta Afore en 7Eleven o Telecomm con tu CURP o llama al 018005222367 ext 3. o, si el cuentahabiente aporta: Hola [[Nombre]], gracias por aportar a tu cuenta InverCap. Ya estas construyendo un mejor futuro con tus Aportaciones Voluntarias. ¡Sigue asi!
New Statement + Small Amounts	Yes	Hola [[Nombre]], ¡Espera tips de Afore via SMS para recordarte de tu Ahorro Voluntario! Estos mensajes te ayudaran a mejorar tu futuro.	Yes	Hola [[Nombre]], viste los tips en tu estado de cuenta Afore? Poco a poco, llega lejos: puedes mejorar tu futuro guardando lo que cuesta un cafecito al dia. Siguiente mensaje de acción (después de unos segundos): Empieza con $10 diarios para tener grandes beneficios a futuro: Aporta a tu cuenta Afore en 7Eleven o Telecomm con tu CURP o llama al 018005222367 opcion 3	Hola [[Nombre]], recuerda que montos chicos mejoran tu futuro: Aporta a tu cuenta Afore en 7Eleven o Telecomm con tu CURP o llama al 01 800 522 2367 opcion 3. o, si el cuentahabiente aporta: Hola [[Nombre]], gracias por aportar a tu cuenta Afore. Ya estas construyendo un mejor futuro con tus Aportaciones Voluntarias. ¡Sigue asi!
New Statement + Individual Goals	Yes	Hola [[Nombre]], ¡Espera tips de InverCap Afore via SMS para recordarte de tu Ahorro Voluntario! Estos mensajes te ayudaran a mejorar tu futuro.	Yes	Hola [[Nombre]], viste los tips en tu Edo. de cuenta Afore? Fija tus metas HOY. Prepara tu futuro, responde: "Familia" "Casa" o "Vacaciones" y obten INFO. Siguiente mensaje de acción (después de unos segundos): Mejora tu futuro y alcanza tus metas HOY con Ahorro Voluntario: Aporta a tu cuenta Afore en 7Eleven o Telecomm con tu CURP o llama al 018005222367 opcion 3	Hola [[Nombre]], recuerda que montos chicos mejoran tu futuro: Aporta a tu cuenta Afore en 7Eleven o Telecomm con tu CURP o llama al 01 800 522 2367 opcion 3. o, si el cuentahabiente aporta: Hola [[Nombre]], gracias por aportar a tu cuenta Afore. Ya estas construyendo un mejor futuro con tus Aportaciones Voluntarias. ¡Sigue asi!
New Statement + Family Security	Yes	Hola [[Nombre]], ¡Espera tips de InverCap Afore via SMS para recordarte de tu Ahorro Voluntario! Estos mensajes te ayudaran a mejorar tu futuro.	Yes	Hola [[Nombre]], viste los tips en tu estado de cuenta InverCap? Actua HOY para mejorar tu futuro y el de tu familia. Guarda para los gastos que vienen. Siguiente mensaje de acción (después de unos segundos): Asegura tu futuro y el de tu familia HOY con Ahorro Voluntario: Aporta a tu cuenta Afore en 7Eleven o Telecomm con tu CURP o llama al 01 800 522 2367 ext3	Hola [[Nombre]], nunca es tarde para asegurar un futuro mejor para ti y tu familia: Aporta en 7Eleven o Telecomm con tu CURP o llama al 01 800 522 2367 ext3. o, si el cuentahabiente aporta: Hola [[Nombre]], gracias por aportar a tu cuenta Afore. Ya estas construyendo un mejor futuro con tus Aportaciones Voluntarias. ¡Sigue asi!

B Robustness of the Main Results

Table S3: Testing Treatment Effects on Contribution Amount (Log Transformed)

Dependent Variable:	Contribution Amount ($) *(Log transformed))*					
	Experimental Period			Post-Experimental Period		
Condition	**Mean**	(1)	(2)	**Mean**	(3)	(4)
Control (Standard Statement)	$114.98	-	-	$93.58	-	-
New Statement	$124.47	.00628	.00411	$123.27	.00639	.00435
		(.00672)	(.00302)		(.00666)	(.00325)
New Statement + Basic Alert	$165.28	.00862	.00268	$120.43	.00599	.00038
		(.0068)	(.00336)		(.0068)	(.00325)
New Statement + Fresh Start	$105.62	.00849	.00359	$95.76	.00716	.00253
		(.00678)	(.00302)		(.00664)	(.00327)
New Statement + Small Amounts	$91.08	-.00074	.00064	$72.76	-.00062	.00068
		(.00638)	(.00325)		(.00629)	(.00370)
New Statement + Individual Goals	$80.20	-.00272	.00517*	$129.78	-.00326	.00418
		(.00626)	(.00308)		(.00618)	(.00342)
New Statement + Family Security	$182.11	.01833**	.01093***	$138.89	.00689*	.01116*
		(.00723)	(.00345)		(.00695)	(.00368)
Prior Contribution (Control)			Y			Y
N	97,149			97,149		

Note: This table reports the estimated relationship of contribution amount as a function of our treatment interventions during our experimental period (October 3 to December 31) and in the two month period *following* our experimental period (January 1-February 28) after all individuals went back to receiving just the standard account statement. We compare treatment interventions relative to the standard account statement. We use the logarithm of one plus the total amount contributed across an individual's contributions. Columns 3 and 4 estimate the same variable but during the post-experimental period. Columns 2 and 4 present results inclusive of a control for prior contribution status. All regressions include a constant. Robust standard errors are used and shown in parentheses. Statistical significance is indicated by *, **, and *** for 10%, 5%, and 1% p-value, respectively, using a two-tailed test.

Table S4: Estimated Treatment Effects Using New Statement as Baseline

Dependent Variable:	Contribution Likelihood		Contribution Amount ($) *(IHS transformed)*)	
Condition	**Mean**	(1)	**Mean**	(2)
New Statement (Baseline)	0.55%	-	$124.47	-
New Statement + Basic Alert	0.59%	-.00011	$165.28	-.00139
		(.00049)		(.00409)
New Statement + Fresh Start	0.60%	.00002	$105.62	-.00091
		(.00046)		(.00381)
New Statement + Small Amounts	0.48%	-.00055	$91.08	-.00604
		(.00046)		(.00387)
New Statement + Individual Goals	0.47%	.00024	$80.20	-.00020
		(.00046)		(.00373)
New Statement + Family Security	0.71%	.00010**	$182.11	.00861**
		(.00047)		(.00408)
N		97,149		97,149

This table reports the estimated relationship of contribution likelihood and contribution amount as a function of our treatment interventions during our experimental period (October 3 to December 31). We compare treatment interventions relative to the new statement. Column (1) estimates the relationship between the treatment interventions versus the new statement on contribution likelihood. Column (2) estimates the relationship between the treatment interventions versus the new statement on total amount contributed during the experimental period using an inverse hyperbolic sine transformation. Positive coefficients represent the behavioral intervention increasing contributions relative to the New Statement, while negative coefficients represent the behavioral intervention decreasing contributions. Results comparing the New Statement versus the Standard Account Statement are presented in Table 2. Robust standard errors are used and shown in parentheses. All regressions include a constant and controls for prior contribution status. Statistical significance is indicated by *, **, and *** for 10%, 5%, and 1% p-value, respectively, using a two-tailed test.

Table S5: Estimated Treatment Effects Using Family Security SMS as Baseline

Dependent Variable:	Contribution Likelihood		Contribution Amount ($) *(IHS transformed))*	
Condition	**Mean**	(1)	**Mean**	(2)
Family SMS	0.71%	-	$182.11	-
Basic Alert SMS *(vs. Family SMS)*	0.59%	-.00110** (.00052)	$165.28	-.00999** (.00440)
Fresh Start SMS *(vs. Family SMS)*	0.60%	-.00097** (.00097)	$105.62	-.00952** (.00414)
Small Amounts SMS *(vs. Family SMS)*	0.48%	-.00154*** (.00049)	$91.08	-.01465*** (.00420)
Individual Goals SMS *(vs. Family SMS)*	0.47%	-.00075 (.00049)	$80.20	-.00881** (.00407)
N		97,149		97,149

This table reports the estimated relationship of contribution likelihood and contribution amount as a function of our treatment interventions during our experimental period (October 3 to December 31). We compare treatment interventions relative to the Family Security SMS. Column (1) estimates the relationship between the treatment interventions versus the Family Security SMS on contribution likelihood. Column (2) estimates the relationship between the treatment interventions versus the Family Security SMS on total amount contributed during the experimental period using an inverse hyperbolic sine transformation. Positive coefficients represent the behavioral intervention increasing contributions relative to the Family Security SMS, while negative coefficients represent the behavioral intervention decreasing contributions. Robust standard errors are used and shown in parentheses. All regressions include a constant and controls for prior contribution status. Statistical significance is indicated by *, **, and *** for 10%, 5%, and 1% p-value, respectively, using a two-tailed test.

Figure S4: Percent Change in Contribution Likelihood Relative to Family SMS

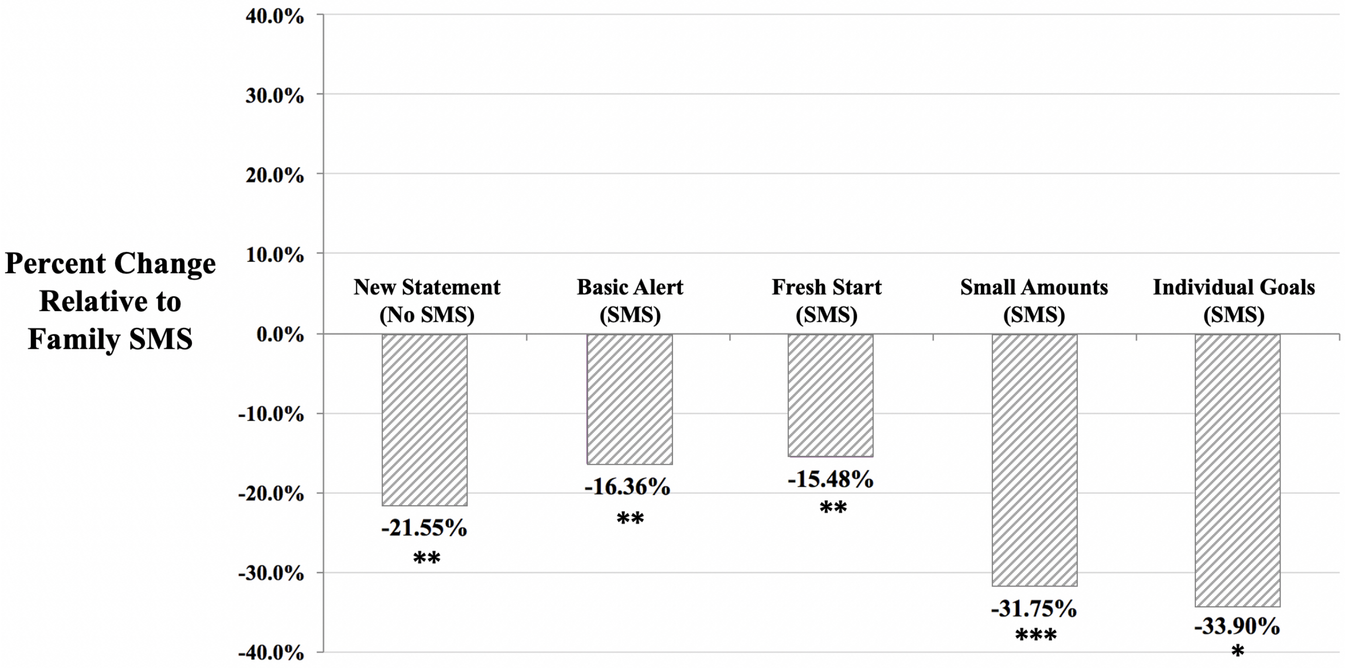

Note: Significance is indicated by *, **, and *** at 10%, 5%, and 1% for a two-tailed test, respectively and is based on a comparison between each experimental group relative to those who received the Family SMS intervention.

C A Guide to Using Recursive Partitioning Approaches: Causal Tree and Causal Forest

In this section, we discuss how researchers can use recursive partitioning to detect heterogeneous treatment effects. One advantage of these approaches is that they are relatively simple to implement, with readily available packages that can be installed in R. Our experience is that Causal Tree, once installed, took roughly five minutes using a Apple Macbook Pro with 16 GB of RAM. Causal Forest took slightly longer, producing results within 30-60 minutes. Models with larger datasets, or more regressors, would likely require more memory or longer computational time to run.

First, researchers will need to install specific packages, if they have not done so already. For Causal Tree, we will need to install the "devtools" package in RStudio, which can be downloaded from github using the website: https://github.com/susanathey/causalTree. The code for the package is as follows:

```
install.packages("devtools")
library(devtools)
installgithub("susanathey/causalTree")
```

It should be noted that other packages may also need to be downloaded for "devtools" to properly run. In our case running the "devtools" package produced the following error message, "There is no package called 'shiny'." Shiny is an R package that makes it easier to build interactive web apps from R. To rectify this issue, we chose Tools –> Install packages, and then downloaded the "shiny" package in RStudio. Researchers may have different dependencies and so should download additional packages accordingly. If you are using Windows, install the correct version of RTools for your version of . For Mac, this may require installing a gcc complier such as Apple's Command Line Tools. Once all packages are downloaded and no errors are indicated, the "devtools" package can be re-run.

Once this is complete, the data can be loaded into R. We then followed the example code provided in the documentation that can be found on this website: https://github.com/susanathey/causalTree/blob/mast
Our approach leveraged the Honesty approach, so followed the code provided in section 5.1, "Honest Estimation," of the documentation. In this approach, the 50% of the data will be used for the training stage while the other 50% is used for the holdout sample. The data that is used

for the 50% holdout, test stage should be saved in a separate location as per the documentation provided by Athey et al. (2016). We specifically used the following:

```
> n <- nrow(simulation.1)
> trIdx <- which(simulation.1$treatment == 1)
> conIdx <- which(simulation.1$treatment == 0)
> trainidx <- c(sample(trIdx, length(trIdx) / 2),
+ sample(conIdx, length(conIdx) / 2))
> traindata <- simulation.1[train_idx,]
> estdata <- simulation.1[-train_idx,]
> honestTree <- honest.causalTree(y   x1 + x2 + x3 + x4,
+ data = traindata,
+ treatment = traindata$treatment,
+ estdata = estdata,
+ esttreatment = estdata$treatment,
+ split.Rule = "CT", split.Honest = T,
+ HonestSampleSize = nrow(estdata),
+ split.Bucket = T, cv.option = "fit",
+ cv.Honest = T)
> opcp <- honestTree$cptable[,1][which.min(honestTree$cptable[,4])]
> opTree <- prune(honestTree, opcp)
> rpart.plot(opTree)
```

In our setting, contribution likelihood was defined as our main outcome measure, “Y," while Age, Gender, and prior contribution status was defined as our x1, x2, and x3. If researchers have more X variables made available for exploring heterogeneous treatment effects, then these additional variables can added to the code after x4 as follows: “honestTree <- honest.causalTree(y   x1 + x2 + x3 + x4 +x5, +x6,....+xN."

It should also be noted that while we used the “CT" criteria for cross validation, there are other evaluation functions made available for computing the cross-validation error. Details for this can be found on github and following the documentation provided by Athey et al. (2016).

Following the example code on the Github website, the resulting tree can be pruned and the dendrogram plotted using the rpart.plot function. Researchers can also specify the minimum number of treated observations and the number of control observations in the leaf to be split by specifying min = n. In our case and following the minimum number of observations specified by Athey and Imbens (2016), we specified the minimum as 2. However, we also tested whether our results changed using different specifications for the min, i.e., 2,3,4,5,6,7,8,9,10,15, and 20. We observed no significant changes in the Causal Tree output regardless of the min specification used. We note that researchers can also specify the minimum and maximum number of buckets, by adding in the line “bucketmin = n, bucketMax = n" following the split.Bucket line entry. Given that we were not interested in constraining the number of buckets, we opted not to specify a minimum or

maximum bucket number. A visual representation of the cutpoints identified in the first, 'training' stage of Causal Tree are portrayed via a dendrogram. In our setting, Figure 4 represents the dendrogram for the first stage of the Causal Tree approach.

Using the 50% holdout sample, we can now move to the estimation and 'test' stage to assess heterogeneous treatment effects. To ensure robust standard errors, researchers should download the 'lmtest' and 'sandwich' packages prior to estimation. Both packages can downloaded by using the Tools–>Install package and typing in 'lmtest' and 'sandwich,' respectively. Once this is done, the researcher can run a regression of the outcome variable on a series of dummy variables corresponding to the different leaves of the resulting tree on the prediction data.

One thing researchers should keep in mind with the Causal Tree procedure is that estimates are based only on a single tree. As a result, prediction error can be high. The exact tree structure obtained from Causal Tree can also be sensitive to the sub-sample of the data used. If we run the procedure using different 50% sub-samples, it is quite possible that some smaller partitions may disappear or be replaced with other small partitions. One approach is to use the Causal Forest procedure, which constructs predictions by averaging over many trees grown on sub-samples of the data. Causal Forest procedures have the advantage of lower prediction variance and greater stability of the estimates(Athey et al., 2019; Breiman, 2001; Wager and Athey, 2018). In addition, as is the case with many statistical tests and machine-learning approaches, researchers should take care to use judgment when making conclusions about heterogeneity in the data. There may be instances where splits or parititons do not align with behavioral patterns or where the partitions occur with such a small sample of the data that meaningful insights may be difficult to glean. Researchers should combine insights gleaned from recursive partitioning techniques along with intuition and other empirical evidence prior to scaling interventions or make accurate predictions broadly.

To run Causal Forest, we used the R package grf. This package is available on the CRAN repository and can be installed in R using this same approach as previous installations. The package description and documentation are available at the website https://cran.r-project.org/web/packages/grf/index.html. The Causal Forest is easier to implement than Causal Tree, because estimation is self-contained a single command (causal_forest). Honesty (sample splitting) is directly incorporated into the causal_forest function, since the Causal Forest is run on many different random sub-samples of the data. Thus, Causal Forest can be run on the entire dataset without a need for splitting the sample before estimation. The syntax for the causal_forest() is as follows. Its first argument, X, is a matrix of independent variables that are used to

estimate treatment effect heterogeneity. In our case, we use age, gender, and prior contribution. The next argument, Y, is the outcome variable - a contribution indicator in our case. The third argument, W, is an indicator for treatment, in our case, if an individual receives the Family Security SMS. The function also allows one to choose a number of optional tuning parameters, as well as the number of CPU threads the algorithm is run on. The tuning parameters can be set automatically through cross-validation by running a function called tune_causal_forest prior to estimation. Our simulation study, presented in Section G of the Supplement, indicated that the results we would expect to see were robust to the tuning parameters, and so we left them at the default values, except the number of trees, which we set to 25,000 to improve statistical efficiency. Again, however, 25,000 trees was likely conservative, as our simulation study indicated the results were robust to the number of included trees.

The causal_forest function returns a large object containing estimation results that can be used to compute conditional average treatment effects. We note that this object can be relatively large in terms of disk space (in our case, it was 2-3 GB). One can compute conditional average treatment effects (CATEs) on subsamples of interest using the function average_treatment_effect included in the grf package, which computes a doubly robust estimate of an average treatment effect. This function takes three arguments. The first is the object returned by the causal_forest function. The second specifies the target sample - in our case, we specified "all" for this option since we wanted to compute average treatment effects for both treatment and control observations. The third option indicates a particular sub-sample of the data to compute the average treatment effect for. This is a vector of TRUE and FALSE values, where TRUE indicates an observation to be included in the CATE calculation. For example, if we wish to compute the CATE for women under 28, we would pass this third argument a vector that is TRUE for all observations in the data where the individual is female and has an age less than or equal to 28. The function returns both the CATE for the indicated sub-sample, as well as a standard error on the CATE, which can be used to compute confidence bounds.

D Causal Tree Estimation Stage Results

Table S6: Contribution Likelihood Results Using the 50% Holdout, 'Test' Sample from the Second Stage Causal Tree (Age Splits Only)

**Age Splits Only**				
Dependent Variable:	Contribution Likelihood (%)			
Coefficient	β	Robust SE	t	P
Family Security SMS × Age ≤ 28	-.00712*	.00397	-1.80	0.072
Family Security SMS × Age ≥ 29	.00296**	.00139	2.13	0.033
Age ≤ 28	.00755**	.00348	2.17	0.030
N	13,878			

**Age & Gender Splits**				
Dependent Variable:	Contribution Likelihood (%)			
Coefficient	β	Robust SE	t	P
Family Security SMS × Age ≤ 28 × Female	-.0151*	.0083	-1.81	0.070
Family Security SMS × Age ≤ 28 × Male	-.0018	.0036	-0.51	0.613
Family Security SMS × Age 29 ≤ 42	.0029*	.0015	1.95	0.051
Family Security SMS × Age ≥ 42	.0034	.0034	1.00	0.318
Age ≤ 28 × Female	.0202***	.0074	2.72	0.007
Age ≤ 28 × Male	.0032	.0033	0.96	0.335
Age 29 ≤ 42	.0028	.0020	1.42	0.157
N	13,878			

Note: This table reports the estimated relationship of contribution likelihood as a function of the age and gender cutpoints implied from the first stage of Causal Forest using the 50% holdout sample. Statistical significance is indicated via *, **, and *** representing 10%, 5%, and 1% for a two-tailed test, respectively. All regressions include a constant.

Table S7: Estimated Treatment Effects Using New Statement as Baseline

Dependent Variable:	Contribution Likelihood (%)		
Condition	**Overall**	Age ≤ 28	Age ≥ 29
New + Basic Alert SMS (*vs. New Statement*)	-.00011	-.00030	-.00007
	(.00049)	(.00147)	(.00051)
New + Fresh Start SMS (*vs. New Statement*)	.00002	-.00090	.00018
	(.00045)	(.00146)	(.00047)
New + Small Amounts SMS (*vs. New Statement*)	-.00055	.00074	-.00077
	(.00046)	(.00138)	(.00048)
New + Individual Goals SMS (*vs. New Statement*)	.00024	.00060	.00018
	(.00046)	(.00130)	(.00049)
New + Family SMS (*vs. New Statement*)	.00099**	-.00058	.00127**
	(.00047)	(.00133)	(.00051)
N	97,149	14,456	82,693

Note: This table reports the estimated relationship of contribution likelihood during our experimental period, comparing the various behavioral intervention relative to sending the redesigned, new account statement alone. We first present the results of the overall main effects, collapsing across all ages. We then present the regression results split by i) those who are 28 years of age and below, and ii) those who are 29 years of age and above. Positive coefficients represent the behavioral intervention performing better relative to sending the new account statement by itself, while negative coefficients represent the behavioral intervention performing worse. The results comparing the New Statement to the Standard Account Statement are not presented as they are presented in Table 3. Robust standard errors are shown in parentheses. All regressions include controls for prior contribution status. Statistical significance is indicated by *, **, and *** for 10%, 5%, and 1% p-value, respectively, using a two-tailed test.

Table S8: Comparing the Family Security SMS to Other SMS Interventions

Dependent Variable:	Contribution Likelihood (%)		
Condition	**Overall**	Age ≤ 28	Age ≥ 29
Family SMS vs. Basic Reminder SMS	.00120**	-.00031	.00134**
	(.00052)	(.00138)	(.00056)
Family SMS vs. Fresh Start SMS	.00097**	.00025	.00108**
	(.00045)	(.00136)	(.00052)
Family SMS vs. Small Amounts SMS	.00153***	-.00135	.00203***
	(.00049)	(.00128)	(.00053)
Family SMS vs. Individual Goals SMS	.00074**	-.00114	.00107**
	(.00049)	(.00120)	(.00054)
N	69,372	10,373	58,999

Note: This table reports the estimated relationship of contribution likelihood and contribution amount (IHS transformed) during our experimental period, comparing the various behavioral intervention relative to the Family Security SMS intervention. We first present the results of the overall main effects, collapsing across all ages. We then present the regression results split by the two overall age buckets implied by the machine-learning approaches: those who are 28 and younger and those who are 29 and older. Negative coefficients represent the SMS behavioral intervention decreasing contributions relative to the Family SMS, while positive coefficients represent the behavioral intervention increasing contribution relative to the Family SMS. All regressions include controls for prior contribution status. Robust standard errors are used and shown in parentheses. Statistical significance is indicated by *, **, and *** for 10%, 5%, and 1% p-value, respectively, using a two-tailed test. All regressions include a constant.

E Test of Heterogeneous Treatment Effect Against Average Treatment Effect

Table S9: Test of HTE versus ATE By Age Splits

	Age Bins Identified by Causal Tree		
Condition	Age ≤ 28	29 ≤ Age ≤ 41	Age ≥ 42
New Statement (*vs. New Statement ATE*)	-.00477*	.00134	.00338
	(.00281)	(.00096)	(.00242)
New + Basic Alert (*vs. Basic Alert SMS ATE*)	-.00432*	.00204	.0008
	(.00287)	(.00099)	(.00188)
New + Fresh Start (*vs. Fresh Start SMS ATE*)	-.00290	.00207	-.00082
	(.00295)	(.00099)	(.00152)
New + Small Amounts (*vs. Small Amounts SMS ATE*)	-.00289	.00051	-.0008
	(.00295)	(.00091)	(.00147)
New + Individual Goals (*vs. Individual Goals SMS ATE*)	-.00552**	.00058	.00164
	(.00272)	(.00092)	(.00208)
New + Family Security (*vs. Family SMS ATE*)	-.00552***	.0037*	.00155
	(.00270)	(.00106)	(.00207)
N	14,456	74,426	8,264

Note: This table presents the conditional average treatment effects of October SMS Treatment on the probability of making a contribution from October 3-December 31, bracketed by age. Coefficients represent comparisons between the heterogeneous treatment effect and the average treatment effect using the coefficients presented in column 2 of Table 2. Robust standard errors are included in parentheses. Statistical significance is indicated by *, **, and *** for 10%, 5%, and 1% p-value, respectively, using a two-tailed test.

Table S10: Test of HTE versus ATE By Age and Gender Splits

Female						
Age Bracket	New Statement	Alert	Fresh Start	Small Amounts	Individual Goals	Family Security
Age ≤ 28	-.0115**	-.01382**	-.01041*	-.01244**	-.017**	-.01456**
	(.03182)	(.00866)	(.05952)	(.0254)	(.00048)	(.00251)
29 ≤ Age ≤ 41	.00254	.00165	.00353*	.0023	.00077	.00529**
	(.2375)	(.46815)	(.08868)	(.1638)	(.93494)	(.04144)
Age ≥ 42	.00764*	.0000	.0000	.0000	.00155	.0000
	(.08207)	(.77613)	(.79206)	(1)	(.80111)	(.45235)

Male						
Age Bracket	New Statement	Alert	Fresh Start	Small Amounts	Individual Goal Security	Family Security
Age ≤ 28	.0000	.00242	.00218	.00368	.00227	.00066
	(.80762)	(.53224)	(.6063)	(.2512)	(.63879)	(.71562)
29 ≤ Age ≤ 41	.000	.00232	.00107	-.00074	.00044	.0026
	(.89885)	(.17093)	(.73888)	(.44092)	(.70052)	(.46367)
Age ≥ 42	-.00204	.0019	-.002	-.00193	.00174	.00349
	(.18513)	(.68297)	(.18188)	(.29538)	(.7997)	(.6227)

Note: This table presents the comparisons between the heterogeneous treatment effect and the average treatment effect using the coefficients presented in Column 2 of Table 2. Robust standard errors are included in parentheses. Statistical significance is indicated by *, **, and *** for 10%, 5%, and 1% p-value, respectively, using a two-tailed test.

F Causal Forest Estimation of Other Interventions

Table S11: Estimated Treatment Effects by Age and Gender (New Statement Alone)

	Estimate		Number of Observations			Estimate		Number of Observations	
Age	Female	Male	Female	Male	Age	Female	Male	Female	Male
20	-0.00361*	4e-05	6	6	40	0.00149	-4e-04	227	255
21	-0.00361*	4e-05	16	18	41	-0.0023	-0.00351	173	204
22	-0.00371*	0	32	53	42	0.02029*	0.00012	140	182
23	-0.00534**	-0.00014	54	79	43	0.0061	-0.00015	143	126
24	-0.02207	-0.00011	92	171	44	-0.00353	-0.00046	104	100
25	-0.00924	0.00013	169	237	45	0.00109	-0.00045	98	99
26	-0.00457	0.0051	317	378	46	0.00142	-0.00062	101	79
27	-0.00403	-0.00017	410	603	47	0.00154	-0.00125	89	79
28	-0.00968*	-0.00302	602	840	48	0.00111	-0.00809	78	64
29	0.00361	-0.00274	736	1058	49	0.00183*	-0.00215	70	44
30	0.00038	0.00056	846	1150	50	0.00167*	-0.00079	81	40
31	-0.00051	-0.00195	903	1284	51	0.00179*	-0.00048	80	28
32	0.00062	0.00367	982	1431	52	0.00212**	-0.00026	52	30
33	0.00186	0.00175	1010	1438	53	0.00334	-9e-05	68	28
34	0.00344	0.00122	1044	1516	54	0.01168	0.00108	58	26
35	0.00331	-0.00048	929	1387	55	0.0058	0.00013	44	24
36	0.00284	0.00233	767	1194	56	0.00725	0.00048	35	21
37	0.00562**	6e-04	520	843	57	0.01027	0.00177	26	29
38	0.00197**	0.00171	324	460	58	0.00541	3e-04	37	21
39	0.00158	-0.00161	270	356	59	0.00468	0.00014	24	21
					60	0.00468	0.00014	9	9

Note: This table depicts the estimated treatment effects and confidence intervals derived from Generalized Random Forest estimation, comparing contribution likelihood for the New Statement intervention relative to the Standard Account Statement during the experimental period. The sample is restricted to those individuals who are in each respective condition, with the sample size totaling to 27,777 individuals. The estimates were derived by using a supplied procedure that estimates the conditional average treatment effect for subsets of the data. Negative coefficients represent the New Statement decreasing contribution likelihood relative to the Standard Account Statement, while positive coefficients represent the New Statement increasing contribution likelihood. All regressions include a constant and controls for prior contribution status. Statistical significance is indicated by *, **, and *** for 10%, 5%, and 1% p-value, respectively, using a two-tailed test.

Table S12: Estimated Treatment Effects by Age and Gender (Basic Alert SMS)

	Estimate		Number of Observations			Estimate		Number of Observations	
Age	Female	Male	Female	Male	Age	Female	Male	Female	Male
20	-0.0046	0.00064	7	2	41	-0.00329	-0.00272	157	209
21	-0.0046	0.00064	12	16	42	0.00059	0.00938	151	171
22	-0.00462	0.00063	20	50	43	0.00037	0.00109	125	140
23	-0.00511*	5e-04	63	74	44	0.00018	0.00091	101	98
24	-0.02295	6e-04	108	157	45	0.00038	0.00052	91	96
25	-0.01082	0.00067	178	240	46	0.00037	0.00013	104	83
26	-0.01047	0.00311	275	387	47	0.00036	-0.00054	99	76
27	-0.00943*	0.0038	422	637	48	6e-05	-0.00661	75	58
28	-0.00699	8e-04	614	828	49	0.00036	-0.00135	97	57
29	0.00348	0.00039	724	1061	50	0.00036	-0.00029	84	34
30	-0.0029	0.00387*	870	1175	51	0.00038	-1e-04	77	24
31	0.0026	0.00132	940	1269	52	0.00035	1e-04	61	40
32	-0.00133	0.00297	971	1425	53	0.00044	0.00021	55	34
33	-0.00025	0.00349*	981	1417	54	0.00047	0.00027	52	25
34	0.00299	0.00179	1014	1538	55	4e-04	0.00028	45	24
35	0.00107	1e-05	935	1422	56	0.00038	3e-04	36	22
36	0.00309	0.0025**	787	1179	57	0.00035	0.00033	30	23
37	0.00405*	0.00279	510	783	58	4e-04	0.00043	26	23
38	0.00314	0.00612*	355	472	59	0.00041	0.00045	26	17
39	0.00085	-0.00048	272	333	60	0.00041	0.00045	10	12
40	0.00067	0.00343	226	244					

Note: This table depicts the estimated treatment effects and confidence intervals derived from Generalized Random Forest estimation, comparing contribution likelihood for the Basic Alert SMS intervention relative to the Standard Account Statement during the experimental period. The sample is restricted to those individuals who are in each respective condition, with the sample size totaling to 27,761 individuals. The estimates were derived by using a supplied procedure that estimates the conditional average treatment effect for subsets of the data. Negative coefficients represent the Basic Alert SMS decreasing contribution likelihood relative to the Standard Account Statement, while positive coefficients represent the Basic Alert SMS increasing contribution likelihood. All regressions include a constant and controls for prior contribution status. Statistical significance is indicated by *, **, and *** for 10%, 5%, and 1% p-value, respectively, using a two-tailed test.

Table S13: Estimated Treatment Effects by Age and Gender (Fresh Start SMS)

	Estimate		Number of Observations			Estimate		Number of Observations	
Age	Female	Male	Female	Male	Age	Female	Male	Female	Male
19	0.01529	0.0046	1	1	40	0.00536	0.0026	215	278
20	0.01529	0.0046	5	6	41	0.00175	-0.00019	165	206
21	0.01529	0.0046	14	13	42	0.00128	0.00026	144	175
22	0.01353	0.00309	24	58	43	0.00097	0.00013	124	135
23	0.00492	0.01047	55	75	44	-0.00402	2e-05	101	104
24	-0.02223	0.00158	96	184	45	0.00772	0.00019	81	94
25	-0.0148	0.00484	156	219	46	0.00157	-0.00017	97	65
26	-0.00986	0.00299	268	391	47	0.00121	-0.00105	91	80
27	-0.00422	0.00331	419	641	48	7e-04	-0.00727	77	58
28	-0.00594	-0.00266	589	843	49	0.00096	-0.00161	98	55
29	-0.00048	-0.00473*	737	1039	50	0.00096	-0.00052	79	34
30	2e-05	0.00406	819	1125	51	0.00102	-0.00021	67	23
31	0.00686*	-0.00047	941	1260	52	0.00097	-5e-05	53	30
32	0.00236	0.00205	956	1426	53	0.00102	-1e-05	53	33
33	0.00293	0.00323	1015	1431	54	0.00099	0	63	26
34	0.00197	0.00144	1043	1558	55	9e-04	2e-05	48	26
35	0.00221	-0.00031	938	1438	56	0.00089	7e-05	40	21
36	0.00165	0.00326*	772	1228	57	0.00086	0.00013	27	23
37	0.00326*	0.00165	504	882	58	0.00085	0.00018	37	21
38	0.00648*	8e-04	329	465	59	0.00086	2e-04	29	17
39	0.0049	-0.001	270	335	60	0.00086	2e-04	14	8

Note: This table depicts the estimated treatment effects and confidence intervals derived from Generalized Random Forest estimation, comparing contribution likelihood for the Fresh Start SMS intervention relative to the Standard Account Statement during the experimental period. The sample is restricted to those individuals who are in each respective condition, with the sample size totaling to 27,784 individuals. The estimates were derived by using a supplied procedure that estimates the conditional average treatment effect for subsets of the data. Negative coefficients represent the Fresh Start SMS decreasing contribution likelihood relative to the Standard Account Statement, while positive coefficients represent the Fresh Start SMS increasing contribution likelihood. All regressions include a constant and controls for prior contribution status. Statistical significance is indicated by *, **, and *** for 10%, 5%, and 1% p-value, respectively, using a two-tailed test.

Table S14: Estimated Treatment Effects by Age and Gender (Small Amounts SMS)

	Estimate		Number of Observations			Estimate		Number of Observations	
Age	Female	Male	Female	Male	Age	Female	Male	Female	Male
19	-	0.00186	0	1	40	0.00065	0.00212	220	248
20	-0.00333	0.00186	6	4	41	-0.00294	-0.00343	182	184
21	-0.00333	0.00186	12	10	42	2e-05	-0.00053	147	172
22	-0.00334	0.00184	32	60	43	-9e-05	-0.00054	122	124
23	-0.00401	0.00855	55	85	44	-0.00469	-0.00061	92	111
24	-0.01576	0.00188	107	160	45	0.00019	-0.00057	93	100
25	-0.0148	0.00785	159	234	46	0.0059	-0.00067	96	83
26	-0.01058	-0.002	292	401	47	9e-04	-0.00139	90	75
27	-0.00286	0.0016	428	623	48	0.00028	-0.008	86	56
28	-0.00992*	0.00175	582	863	49	0.00055	-0.00231	89	48
29	-0.00241	-0.0047*	735	1055	50	0.00056	-0.00107	68	39
30	0.00096	0.00107	849	1181	51	0.00047	-0.00073	79	25
31	0.00106	-0.00153	922	1275	52	0.00043	-0.00056	60	29
32	0.00115	-0.00024	951	1404	53	0.00046	-0.00051	64	35
33	-0.00128	-0.00135	1022	1443	54	0.00048	-0.00046	68	26
34	0.00413	8e-05	1028	1511	55	5e-04	-0.00041	39	22
35	0.00385	0.00014	960	1398	56	0.00046	-0.00036	38	24
36	0.00362	0.00084	750	1169	57	0.00041	-0.00032	27	24
37	0.00258	0.00115	528	844	58	0.00042	-0.00027	28	21
38	0.00279	0.00028	352	466	59	0.00043	-0.00026	27	23
39	0.00084	0.00024	281	339	60	0.00043	-0.00026	9	9

Note: This table depicts the estimated treatment effects and confidence intervals derived from Generalized Random Forest estimation, comparing contribution likelihood for the Small Amounts SMS intervention relative to the Standard Account Statement during the experimental period. The sample is restricted to those individuals who are in each respective condition, with the sample size totaling to 27,779 individuals. The estimates were derived by using a supplied procedure that estimates the conditional average treatment effect for subsets of the data. Negative coefficients represent the Small Amounts SMS decreasing contribution likelihood relative to the Standard Account Statement, while positive coefficients represent the Small Amounts SMS increasing contribution likelihood. All regressions include a constant and controls for prior contribution status. Statistical significance is indicated by *, **, and *** for 10%, 5%, and 1% p-value, respectively, using a two-tailed test.

Table S15: Estimated Treatment Effects by Age and Gender (Individual Goals SMS)

	Estimate		Number of Observations			Estimate		Number of Observations	
Age	Female	Male	Female	Male	Age	Female	Male	Female	Male
20	-0.00543**	0.00041	6	5	41	-0.0034	0.00062	167	194
21	-0.00543**	0.00041	13	11	42	0.00026	0.00445	149	186
22	-0.00545**	4e-04	27	62	43	8e-05	0.00141	119	131
23	-0.00662**	0.00044	53	75	44	-0.00445	0.00116	98	107
24	-0.02402	0.00063	101	172	45	0.00606	0.00123	85	101
25	-0.01672**	0.00332	162	255	46	0.00114	0.00118	106	64
26	-0.01514**	0.003	281	376	47	0.00094	0.00101	104	72
27	-0.00854	0.00315	424	625	48	0.00079	-0.00061	77	59
28	-0.01084*	-0.00147	582	853	49	0.00112	0.00062	92	43
29	3e-05	-0.00263	738	1040	50	0.00132	0.00068	70	35
30	-0.00079	0.00021	886	1202	51	0.00272	0.00067	60	30
31	-0.00109	-0.00151	871	1283	52	0.00859	0.00119	59	40
32	6e-04	-0.00012	951	1400	53	0.00224	0.00067	60	38
33	0.00064	-0.00119	1002	1468	54	0.00126	0.00065	61	30
34	-0.00167	-0.00029	988	1509	55	0.00095	0.00067	46	25
35	-0.00064	0.00285	954	1437	56	0.00083	0.00076	37	18
36	0.00148	0.00219	793	1186	57	0.00073	0.00078	28	16
37	0.00353	0.00206	526	842	58	0.00068	0.00079	31	18
38	0.00133	0.0026	332	487	59	0.00067	0.00079	33	18
39	0.00327	0.00118	282	360	60	0.00067	0.00079	9	11
40	0.00446	0.00747	214	242					

Note: This table depicts the estimated treatment effects and confidence intervals derived from Generalized Random Forest estimation, comparing contribution likelihood for the Individual Goals SMS intervention relative to the Standard Account Statement during the experimental period. The sample is restricted to those individuals who are in each respective condition, with the sample size totaling to 27,803 individuals. The estimates were derived by using a supplied procedure that estimates the conditional average treatment effect for subsets of the data. Negative coefficients represent the Individual Goals SMS decreasing contribution likelihood relative to the Standard Account Statement, while positive coefficients represent the Individual Goals SMS increasing contribution likelihood. All regressions include a constant and controls for prior contribution status. Statistical significance is indicated by *, **, and *** for 10%, 5%, and 1% p-value, respectively, using a two-tailed test.

Table S16: Estimated Treatment Effects by Age and Gender (Family Security SMS)

	Estimate		Number of Observations			Estimate		Number of Observations	
Age	Female	Male	Female	Male	Age	Female	Male	Female	Male
19	-0.00497**	-	1	0	40	0.00512	0.00525*	224	262
20	-0.00497**	0.00036	8	4	41	-0.00203	0.00645	169	186
21	-0.00497**	0.00036	14	11	42	0.00128	0.00684	154	183
22	-0.00501**	0.00034	30	72	43	0.00142	0.01327	121	132
23	-0.00702**	0.00038	51	86	44	-0.00385	0.00192	107	114
24	-0.02387	6e-04	93	179	45	0.00076	0.00168	85	88
25	-0.01663*	0.00335	165	253	46	0.00109	0.00132	86	69
26	-0.01498**	0.00232	280	405	47	0.00113	0.00053	97	68
27	-0.00989*	0.00036	419	615	48	0.00094	-0.00656	86	63
28	-0.00491	-0.00079	600	812	49	0.00146	-0.00067	86	53
29	0.00362	-2e-05	695	1063	50	0.00158	0.00041	77	42
30	0.00133	0.00273	850	1213	51	0.00262	0.00064	72	29
31	0.00249	-0.00026	907	1248	52	0.00924	0.00172	57	33
32	0.00286	0.00107	950	1419	53	0.00309	0.001	52	36
33	0.00835**	0.00145	1052	1439	54	0.00177	0.00101	63	28
34	0.00558*	0.00384*	1038	1543	55	0.00148	0.00104	45	24
35	0.00444**	0.0027	930	1399	56	0.00138*	0.00109	41	19
36	0.00367*	0.00545**	776	1183	57	0.00131*	0.00116	28	24
37	0.00413**	0.00479	514	816	58	0.00132*	0.00123	28	24
38	0.00903*	0.00635**	352	438	59	0.00135*	0.00126	27	19
39	0.0026**	0.00283	272	344	60	0.00135*	0.00126	7	8

Note: This table depicts the estimated treatment effects and confidence intervals derived from Generalized Random Forest estimation, comparing contribution likelihood for the Family Security SMS intervention relative to the Standard Account Statement during the experimental period. The sample is restricted to those individuals who are in each respective condition, with the sample size totaling to 27,755 individuals. The estimates were derived by using a supplied procedure that estimates the conditional average treatment effect for subsets of the data. Negative coefficients represent the Family Security SMS decreasing contribution likelihood relative to the Standard Account Statement, while positive coefficients represent the Family Security SMS increasing contribution likelihood. All regressions include a constant and controls for prior contribution status. Statistical significance is indicated by *, **, and *** for 10%, 5%, and 1% p-value, respectively, using a two-tailed test.

G Identifying Heterogeneity Using Causal Forest: Artificial Data Experiment & Analyses

We estimate a Causal Forest using the Generalized Random Forest approach of Wager and Athey (2018) on the family treatment relative to the control. We once again use the proportion making a savings contribution as our main outcome variable of interest. Figure 5 presents the plots of the resulting conditional average treatment effects by age and gender. We use gender, age, and prior contribution status as our potential moderators of interest.[9] We can directly apply Causal Forest to the entire dataset without explicitly specifying training and estimation data, because the underlying trees used to compute treatment effects are honest: For every observation in the data, the treatment effect is estimated using trees grown on other random subsamples of the data that do not contain that observation.[10]

It can be seen in Figure 5 that for women, there is an increase in the effectiveness of the Family Security treatment (from negative to positive) around age 28, and the positive effects diminish just after age 40. For men, we see a similar increase around age 28 and a decrease after age 40, although the predicted effects are not negative for younger men. Overall, the results suggest that there are significant heterogeneous treatment responses. Moreover, the results not only confirm the validity of the Causal Tree results but can also help determine whether there are heterogeneous outcomes across all treatment conditions as a function of these age brackets and gender or whether this is specific to the Family Security SMS in particular.

For comparison, we present the estimated treatment effects from using the Causal Forest approach for all treatments for all values of age and gender. This can be found in Appendix F, Tables S11 through Tables S16.

We evaluate the sensitivity of estimated treatment effects from the Causal Forest estimator to tuning parameters using a simulation exercise. We construct a simulated dataset which mimics the dataset used for estimation in the following way: First, we take a population of $N = 25,000$ simulated individuals and split individuals into treatment and control groups with probability 0.5. Next, we randomly assign individuals to a) either gender with probability 0.5, b) an age between 20 and 65, putting equal weight on each year, and

[9]Although we include prior contribution status as an independent variable in the Causal Forest, we found it does not split on it. Thus, we only present plots of CATEs for age and gender.

[10]The confidence intervals shown in the plots are produced by the estimation procedure, and are based on the infinitesimal jackknife estimator. We use the supplied R package called 'grf' and use the default values and 5,000 trees for our tuning parameters. We also present the results of several simulation studies to show that a dataset of our size using a similar data generating process for estimated heterogeneous treatment effects produce estimated effects are robust to the number of trees and the tuning parameter values.

c) to be a prior contributor with probability 0.05. We assume the individual's outcome is determined by the equation:

$$y_i = 0.01 + T_i(\delta_1 \mathbf{1}\{age_i \leq 30\} + \delta_2 \mathbf{1}\{age_i > 30 \& age_i \leq 45\} + \delta_3 \mathbf{1}\{age_i > 45\}) + 0.8 prior_i + \epsilon_i,$$

where $\epsilon_i \sim N(0, 0.0064)$ is i.i.d. across individuals, and the effects of the treatment by age are $\delta_1 = -0.01$, $\delta_2 = 0.01$, and $\delta_3 = 0$. age_i represent individual i's age, and $prior_i$ is an indicator variable with 1 indicating that individual i has made a prior contribution. After generating the artificial data, we compute estimated treatment effects using Causal Forest for each individual using (1) the default settings with 5,000 trees, (2) automatic tuning procedure with 5,000 trees, and (3) the default settings with 25,000 trees. In Table S17, we show the conditional average treatment effects for each estimation run for the age bins corresponding to where the parameters change. The results show that the parameter estimates are close to the truth in all cases, and the true parameters are well within the 95% confidence bounds implied by the estimated standard errors. We do not perform a full Monte Carlo study that includes coverage rates, as the computational burden of running the estimation once is high.

Table S17: Conditional Average Treatment Effects in Simulation Study

Age Bracket	Default Settings (5,000 Trees)	Tuned Parameters (5,000 Trees)	Default Settings (25,000 Trees)	True Values
Age ≤ 30	-0.0098	-0.0097	-0.0096	-0.01
	(0.0023)	(0.0021)	(0.0023)	
30 < Age ≤ 45	0.0089	0.0089	0.009	0.01
	(0.0019)	(0.0018)	(0.0018)	
Age > 45	-6e-04	-6e-04	-5e-04	0
	(0.0016)	(0.0015)	(0.0016)	

H Identifying Heterogeneity Using OLS Approaches

Table S18: Identifying Heterogeneity in Family Security SMS Using OLS

Coefficient	Gender (Only)	Gender & Linear Age	Gender & Quadratic Age	Gender & Age Bins
Treat	.00184***	.00007	-.00187	.00304***
	(.00061)	(.00132)	(.00233)	(.00109)
Female × Treat	-.00053	.00000	-.00137	-.00301
	(.00095)	(.00173)	(.00366)	(.00217)
Trend × Treat		.00012	.00038	
		(.00009)	(.00027)	
Female × Trend × Treat		-.00004	.0001	
		(.00010)	(.00038)	
Trend^2 × Treat			-.00001	
			(.00001)	
Female × Trend^2 × Treat			.0000	
			(.00001)	
Age 31-40				.00167
				(.0010)
Age 41-50				.00206
				(.0010)
Age 50+				.00138
				(.00085)
Age 31-40 × Treat				-.00178
				(.00134)
Age 41-50 × Treat				-.000479
				(.00236)
Age 50+ × Treat				-.00304***
				(.00109)
Female × Age 31-40 × Treat				.00374
				(.00248)
Female × Age 41-50 × Treat				-.00017
				(.00303)
Female × Age 50+ × Treat				.00762
				(.00445)

Note: This table reports the estimated relationship of contribution likelihood as a function of the Family Security treatment intervention, and various treatment by demographic interaction terms during our experimental period (October 3 to December 31). We compare the main effect of the treatment as well as the interaction terms relative to our baseline condition: the standard, control account statement. The sample is restricted to individuals in the control or family treatment conditions, and the total sample size is 27,755 individuals. Robust standard errors are used and shown in parentheses. Statistical significance is indicated by *, **, and *** for 10%, 5%, and 1% p-value, respectively, using a two-tailed test. All regressions include a constant as well as controls for prior contribution status and the main effects of the variables used for interactions (a gender indicator, age trends/age bucket indicators).

Table S19: Identifying Heterogeneity Using OLS with 5-Year Age Bins

Coefficient	**Men Only** (*Baseline = Age ≤ 24*)	**Women Only** (*Baseline = Age ≤ 24*)
Family SMS	.00102**	.00187
	(.00046)	(.0019)
Age 26-30	-.00156*	.00152
	(.00087)	(.00241)
Age 31-35	.00051	.00145
	(.00056)	(.00197)
Age 36-40	-.00035	.00014
	(.00090)	(.00224)
Age 41-45	.00048	.00238
	(.00049)	(.00202)
Age 46-50	.00117	.00133
	(.00119)	(.00183)
Age 51-55	.0000	.00133
	(.0000)	(.00183)
Age 56-60	.0000	.00133
	(.0000)	(.00183)
Family SMS × Age 26-30	-.00239**	-.00137
	(.0012)	(.00284)
Family SMS × Age 31-35	-.00064	-.00005
	(.00101)	(.00206)
Family SMS × Age 36-40	.00356	.00183
	(.00262)	(.00335)
Family SMS × Age 41-45	.00314	-.00292
	(.00300)	(.00209)
Family SMS × Age 46-50	-.0022*	-.00187
	(.00131)	(.0019)
Family SMS × Age 51-55	-.00102**	-.00022
	(.00046)	(.00246)
Family SMS × Age 56-60	-.00102**	-.00187
	(.00046)	(.00190)
N	16,046	11,709

Note: This table reports the estimated relationship of contribution likelihood as a function of the main effect of the Family Security SMS intervention, 5-year age intervals, and various treatment by 5-year age interval interaction terms. We estimate these effects during our experimental period (October 3 to December 31). We compare the main effect of the treatment as well as the interaction terms relative to our baseline condition: the standard, control account statement. Column 1 restricts the sample to men, while column 2 restricts the sample to women. Robust standard errors are used and shown in parentheses. *, **, and *** indicate significance at 10%, 5%, and 1% for a two-tailed test. All regressions include a constant as well as controls for prior contribution status.

Table S20: Estimating Treatment Effect by Age and Gender Using OLS (All Treatments)

Women						
Age Bracket	New Statement	Alert	Fresh Start	Small Amounts	Individual Goals	Family Security
Age ≤ 28	-.0113**	-.01361**	-.01060*	-.01225**	-.01691***	-.01455***
	(.00567)	(.00543)	(.00581)	(.00561)	(.00509)	(.00536)
29 ≤ Age ≤ 41	.00262	.00168	.00359**	.00231	.00080	.00540***
	(.0016)	(.00153)	(.00167)	(.00158)	(.00147)	(.00179)
Age ≥ 42	.00757*	.00001	.00006	.0006	.00159	.00001
	(.00489)	(.00210)	(.0022)	(.00214)	(.00263)	(.00214)

Men						
Age Bracket	New Statement	Alert	Fresh Start	Small Amounts	Individual Goals	Family Security
Age ≤ 28	-.00004	.00243	.00222	.00376	.00220	.00059
	(.00265)	(.00300)	(.00295)	(.00314)	(.00294)	(.00271)
29 ≤ Age ≤ 41	.00048	.00227*	.00103	-.00076	.00042	.00257**
	(.00118)	(.00130)	(.00121)	(.00109)	(.00117)	(.00131)
Age ≥ 42	-.00191	.00183	-.00191	-.00191	.00194	.00372
	(.00205)	(.00326)	(.00205)	(.00191)	(.00332)	(.00376)

Note: This table depicts the estimated treatment effects using OLS. We compare all intervention to the control group on the likelihood to make a contribution during the experimental period. Negative coefficients represent the behavioral intervention performing worse relative to the Control condition, while positive coefficients represent the behavioral intervention performing better. All regressions include controls for prior contribution status. Robust standard errors are used and shown in parentheses. *, **, and *** indicate significance at 10%, 5%, and 1% for a two-tailed test.

I Measuring Persistence Post-Intervention

Table S21: Measuring Treatment Effects During & Post-Experiment

Dependent Variable:	Contribution Likelihood (%) *(First two months of experiment)*		Contribution Likelihood (%) *(Two months post-experiment)*	
Condition	(1)	(2)	(3)	(4)
New Statement	.00095	.00094**	.00059	.00058
	(.00085)	(.00042)	(.00086)	(.00047)
New Statement + Basic Alert	.00124	.00076	.00059	.00014
	(.00087)	(.00047)	(.00088)	(.00048)
New Statement + Fresh Start	.00116	.00075*	.00094	.00056
	(.00086)	(.00043)	(.00088)	(.00051)
New Statement + Small Amounts	.00008	.00017	.00008	.00016
	(.00081)	(.00042)	(.00084)	(.00052)
New Statement + Individual Goals	-.00014	.00096**	-.00043	.0006
	(.00081)	(.00043)	(.00082)	(.0005)
New Statement + Family Security	.00218**	.00164***	.00168*	.00117***
	(.00090)	(.00043)	(.00091)	(.00053)
Prior Contribution (Control)		Y		Y
N	97,149	97,149	97,149	97,149

Note: This table reports the estimated relationship of contribution likelihood as a function of our treatment interventions. Columns (1) and (2) estimate the relationship between our treatments interventions versus a standard, control statement on contribution likelihood in the first two months of our experimental period. Columns (3) and (4) estimate the relationship between our treatments interventions versus a standard, control statement on contribution amount in the two-month period *following* our experimental period after all individuals were all sent the standard account statement without any follow-up SMS interventions. We compare treatment interventions relative to the group who were given the standard, control account statement during the main experimental period. In specifications (2) and (4), we present our results inclusive of a control for prior contribution status indicating whether a contribution was made in the year prior to the intervention for each period of interest. Robust standard errors are used and shown in parentheses. Statistical significance is indicated by *, **, and *** for 10%, 5%, and 1% p-value, respectively, using a two-tailed test. All regressions include a constant.

Table S22: Measuring Persistence: Estimating Treatment Effects By Age Intervals (Post-Intervention)

Dependent Variable:	Contribution Likelihood (%)			
Condition	**Overall**	Age ≤ 28	29 ≤ Age ≤ 41	Age ≥ 42
New Statement	.00058	-.00104	.00108**	-.00055
	(.00047)	(.00135)	(.00054)	(.00146)
New + Basic Alert	.00014	-.00179	.00067	-.00009
	(.00048)	(.00170)	(.0006)	(.00141)
New + Fresh Start	.00056	-.00085	.00104	-.00131
	(.00051)	(.00156)	(.00055)	(.00166)
New + Small Amounts	.00016	-.00073	.00054	-.00168
	(.00052)	(.0016)	(.00058)	(.00134)
New + Individual Goals	.00006	.00093	.00073	-.00121
	(.00005)	(.00159)	(.00056)	(.00125)
New + Family Security	.00117***	-.00212	.00201***	-.00045
	(.00053)	(.00145)	(.0006)	(.00135)
N	97,149	14,456	74,429	8,264

Note: This table reports the estimated relationship of contribution likelihood in the period post intervention (January 1 to February 28), comparing the various behavioral intervention relative to the Standard Account Statement. We first present the results of the overall main effects, collapsing across all ages. We then present the regression results split by our three age buckets implied by the machine-learning approaches: those who are 28 and younger, those between 29 and 41, and those who are 42 and older. Note: After the conclusion of the experimental period, individuals were sent the standard account statement and no additional follow-up texts regardless of treatment. Robust standard errors are used and shown in parentheses. Statistical significance is indicated by *, **, and *** for 10%, 5%, and 1% p-value, respectively, using a two-tailed test. All regressions include a constant and controls for prior contribution status.

Table S23: Contribution Likelihood Post-Experiment Using the Family SMS as Baseline

Dependent Variable:	Contribution Likelihood (%)		
Condition	**Overall**	Age ≤ 28	Age ≥ 29
Standard Statement (*vs. Family SMS*)	-.00166***	.00144	-.00208***
	(.00047)	(.00125)	(.00051)
New Statement (*vs. Family SMS*)	-.00092*	.0006	-.00118**
	(.00047)	(.00133)	(.00050)
New + Basic Alert (*vs. Family SMS*)	-.00110 **	.00027	-.00134**
	(.00052)	(.00139)	(.00056)
New + Fresh Start (*vs. Family SMS*)	-.00097 **	-.00033	-.00108**
	(.00049)	(.00137)	(.00052)
New + Small Amounts (*vs. Family SMS*)	-.00097 ***	.00131	-.00204***
	(.00049)	(.00128)	(.00053)
New + Individual Goals (*vs. Family SMS*)	-.00153	.00118	-.00108**
	(.00049)	(.00120)	(.00054)
N	97,149	14,456	82,693

Note: This table presents the estimates of the relationship between our experimental groups versus the Family Security SMS on contribution likelihood in the two month period *following* our experimental period, after all individuals went back to the standard account statement. Robust standard errors are used and shown in parentheses. Robust standard errors are used and shown in parentheses. All regressions include a constant and controls for prior contribution status. Statistical significance is indicated by *, **, and *** for 10%, 5%, and 1% p-value, respectively, using a two-tailed test.

Table S24: Contribution Amount Post-Experiment Using the Family SMS as Baseline

Dependent Variable:	Contribution Amount ($) *(IHS transformed)*)		
Condition	**Overall**	Age ≤ 28	Age ≥ 29
Standard Statement (*vs. Family SMS*)	-.01049**	.00783	-.01376***
	(.00435)	(.01137)	(.00474)
New Statement (*vs. Family SMS*)	-.00365	.00805	-.00574
	(.00422)	(.01021)	(.00463)
New + Basic Alert (*vs. Family SMS*)	-.007852 *	-.00221	-.00888 *
	(.00456)	(.01240)	(.00491)
New + Fresh Start (*vs. Family SMS*)	-.00525	-.00033	-.00832*
	(.00433)	(.00137)	(.00464)
New + Small Amounts (*vs. Family SMS*)	-.01016**	.00793	-.01341***
	(.00451)	(.01251)	(.00482)
New + Individual Goals (*vs. Family SMS*)	-.00575	.01788	-.00992**
	(.00429)	(.01152)	(.00462)
N	97,149	14,456	82,693

Note: This table presents the estimates of the relationship between our experimental groups versus the Family Security SMS on total contribution amount (using an inverse hyperbolic sine transformation) in the two month period *following* our experimental period, after all individuals went back to the standard account statement. Robust standard errors are used and shown in parentheses. Robust standard errors are used and shown in parentheses. All regressions include a constant and controls for prior contribution status. Statistical significance is indicated by *, **, and *** for 10%, 5%, and 1% p-value, respectively, using a two-tailed test.
